# Molecular evidence of *Echinococcus canadensis* (G6/G7) predominance in Mongolian livestock and its implications for control

**DOI:** 10.1371/journal.pntd.0014433

**Published:** 2026-06-15

**Authors:** Bolor Bold, Peter S. Andrus, Chimedtseren Bayasgalan, Davaasuren Nergui, Battsetseg Badmaa, Yilin Cao, Xuan Su, Xiaojin Mo, Nyamdavaa Guugandaa, Ning Xiao, Ting Zhang, Xiao-Nong Zhou

**Affiliations:** 1 School of Global Health, Chinese Center for Tropical Diseases Research, Shanghai Jiao Tong University School of Medicine, Shanghai, China; 2 National Institute of Parasitic Diseases, Chinese Center for Disease Control and Prevention (Chinese Center for Tropical Diseases Research); National Key Laboratory of Intelligent Tracking and Forecasting for Infectious Diseases; NHC Key Laboratory of Parasite and Vector Biology, WHO Collaborating Centre for Tropical Diseases, Shanghai, China; 3 National Center for Zoonotic Diseases, Ministry of Health, Ulaanbaatar, Mongolia; 4 Academy of Pharmacy, Xi’an Jiaotong-Liverpool University, Suzhou, China; 5 Department of Infectious Diseases and Microbiology, School of Veterinary Medicine, Mongolian University of Life Sciences, Ulaanbaatar, Mongolia; 6 State Quality Control Laboratory for Veterinary Drugs, Ulaanbaatar, Mongolia; Washington University in St Louis School of Medicine, UNITED STATES OF AMERICA

## Abstract

**Background:**

Cystic echinococcosis (CE), caused by *Echinococcus granulosus sensu lato* (*E. granulosus s.l.*), is a major zoonotic disease causing substantial health and economic losses, particularly in pastoral communities. WHO and WOAH recommend combining regular dog deworming with sheep vaccination (EG95) for control. However, genotype data are important for vaccination planning, as EG95 mainly targets G1/G3 in sheep and evidence of protection against other genotypes is limited. In Mongolia, molecular data from livestock, especially sheep, remain scarce despite the large sheep population. We therefore aimed to determine the genotype distribution of *E. granulosus s.l.* among livestock, particularly sheep, across Mongolia’s endemic provinces.

**Methods:**

From August to December 2024, we conducted an abattoir-based cross-sectional survey in four provinces (Umnugobi, Bayankhongor, Dundgobi, and Tuv). Sheep, goats, and camels were examined for hydatid cysts. DNA was extracted, the *COX1* gene was amplified by PCR and sequenced using the Sanger method, and published diagnostic *COX1* sites were used to refine G6/G7 assignments; phylogenetic and haplotype analyses were performed with regional reference data.

**Results:**

Cysts were detected in 2.5% (115/4,578) of animals, and 0.31% (14/4,578) were molecularly confirmed as *E. granulosus s.l.* Among them, 93% (13/14; 10 sheep, 2 goats, 1 camel) were *Echinococcus canadensis* (G6/G7) and 7% (1/14; 1 sheep) were *Echinococcus granulosus s.s.* (G1/G3*)*. Five *COX1* haplotypes were identified: four *E. canadensis* haplotypes (H1–H4) and one *E. granulosus s.s.* haplotype (H5). Haplotypes H1–H3 were consistent with G6, whereas H4 was consistent with G7a. These haplotypes grouped with reference isolates from Mongolia and neighbouring countries, as well as from other endemic regions included in the network.

**Conclusions:**

This study strengthens evidence that *E. granulosus s.l.* infections in Mongolian livestock are predominantly caused by *Echinococcus canadensis* G6/G7 and shows, for the first time, that this taxon predominated among successfully genotyped isolates from sheep in the surveyed areas. These findings support genotype-informed surveillance for vaccination planning, particularly in mixed-genotype settings, while underscoring the importance of dog deworming and safe offal disposal. Our findings further clarify CE transmission patterns in Mongolia and the wider region, highlight the transboundary nature of CE, and support the need for coordinated cross-border surveillance and control.

## Author summary

Cystic echinococcosis (CE) is a parasitic disease transmitted between dogs and livestock that can cause serious illness in people. It is common in Mongolia, where pastoral livelihoods rely on large animal herds and dogs often roam freely. The World Health Organization and the World Organisation for Animal Health recommend combining regular dog deworming with sheep vaccination (EG95) to reduce transmission. However, EG95 was developed mainly for the G1/G3 genotype of the parasite, so understanding which genotype circulates locally is important for deciding how vaccination might fit into control programmes.

We surveyed sheep, goats, and camels from four endemic provinces in Mongolia and used DNA testing to identify the *Echinococcus* species and genotype groups present. Most confirmed infections were caused by *Echinococcus canadensis* (G6/G7), including isolates from sheep, while only one sample belonged to *Echinococcus granulosus s.s* (G1/G3).

These findings show that CE transmission in Mongolian livestock, including sheep, might be largely driven by G6/G7. This supports genotype-informed control planning and highlights the need to carefully evaluate the role of EG95 in G6/G7-dominated or mixed-genotype settings. In addition, our results suggest that non-vaccine measures, such as dog deworming and improved slaughterhouse management, may be particularly important in Mongolia and other mixed-genotype settings.

## Introduction

Cystic echinococcosis (CE), caused by the *Echinococcus granulosus sensu lato* (*E. granulosus s.l.*) complex, imposes an estimated annual burden of ~184,000 disability-adjusted life years (DALYs) and ≈US$3 billion in combined human health-care costs and livestock-sector losses [1,2]. CE is recognised by the World Health Organization (WHO) as one of the priority neglected tropical diseases and affects mainly pastoral and nomadic communities worldwide [3,4].

A well-established dog–livestock cycle of *E. granulosus s.l.* persists in pastoral systems and is difficult to interrupt [5]. Adult tapeworms develop in the dog’s small intestine and eggs are shed through feces, contaminating pastures, water, soil, vegetation, and animal surfaces [6]. Grazing livestock become infected by ingesting these eggs, leading to CE development mainly in the liver and lungs. The cycle is completed when dogs gain access to raw viscera or offal (e.g., liver or lungs from slaughtered animals) infected with hydatid cysts that may contain protoscoleces. These protoscoleces mature into adult tapeworms in the intestine and shed eggs again in feces [5]. Humans become infected by ingesting eggs shed in canid feces, most often via hand-to-mouth contact after exposure to contaminated sources, and can develop CE [6]. Human CE can cause substantial health and economic burden: cyst growth may lead to organ compression and obstruction, while rupture can cause anaphylaxis, dissemination, and relapse, often requiring repeated, complicated surgery and long-term care [7,8].

Several interventions can target key steps in the transmission cycle to interrupt spread. [9]. A key measure is regular praziquantel deworming of dogs, which effectively reduces egg shedding but requires sustained, high-coverage delivery because reinfection can occur where dogs have access to infected offal [10]. Improved slaughter and offal management that restricts dog access to abattoirs and ensures safe disposal of infected organs can substantially reduce dog reinfection, although implementation can be costly and may require policy and infrastructure changes. Health education and behaviour change, including hand hygiene and avoiding feeding raw offal to dogs can reduce human exposure, but progress is often slow and requires sustained effort over time, especially in rural pastoral settings [9,11].

Another approach is vaccination. Currently, there is no vaccine for dogs, but the EG95 vaccine for sheep has shown promising results by reducing cyst establishment in lambs and, over time, lowering the amount of infected offal available to dogs [12,13]. EG95 has shown high protection in experimental and field trials in several countries, including Australia, Argentina, Chile, and China [14–16]. Both WHO and the World Organisation for Animal Health (WOAH) recommend an integrated strategy that combines sheep vaccination with dog deworming to accelerate elimination [17].

A key consideration before implementing EG95 is whether the dominant parasite genotypes circulating in livestock, particularly sheep, are compatible with the vaccine [18]. Under current taxonomy, these genotypes within the *E. granulosus s.l.* complex are commonly reported using species names, including *E. granulosus sensu stricto* (*E. granulosus s.s*.) (G1/G3), *Echinococcus equinus* (G4), *Echinococcus ortleppi* (G5), and *Echinococcus canadensis* (*E. canadensis*) (G6/G7 and G8/G10), which differ in host associations, transmission cycles, and ecological characteristics [19–22]. Alvarez et al. (2014) found that approximately 99% of human CE cases are attributed to two groups: *E. granulosus s.s.* (G1/G3), typically maintained in dog–sheep cycles and accounting for 88% of cases, and *E. canadensis* (G6/G7), more often associated with dog–camel/goat/pig cycles and contributing 11% [23]. The EG95 vaccine is a recombinant oncosphere antigen developed from parasites of the G1/G3 genotype group. However, antigenic variation has been reported in G6/G7 parasites, which may affect vaccine effectiveness against these genotypes. Its performance in G6/G7-dominated transmission settings remains insufficiently studied [9,15].

In Mongolia, roughly one-third of the population practices nomadic pastoralism, managing ~60 million sheep, goats, cattle, horses and camels, with widespread dog ownership and frequent informal slaughter. Following veterinary sector privatization in the 1990s, earlier control programs for CE ceased [24–26]. Robust nationwide estimates of CE prevalence in livestock are not available, as CE is not systematically captured within the existing livestock surveillance system; published data are limited to a few localized abattoir-based surveys, mainly in camels [27,28]. Reported human surgical incidence is 2.2 per 100,000 annually, with adjustments suggesting a substantially higher incidence (up to ~ 8-fold) [29–31].

Molecular data on *E. granulosus s.l.* remain scarce in Mongolia, particularly in livestock. Available human studies have reported *E. granulosus s.l.* (G1/G3), *E. canadensis* (G6/G7, and G10), with *E. canadensis* comprising approximately 93% of detections are in paediatric cases [32,33]. In wildlife, G6/G7 has been reported in foxes and G10 in wolves, and a recent suburban stray-dog survey identified G6/G7 as the main genotype [34]. Although these findings show evidence of substantial circulation of *E. canadensis*, data from livestock—central to transmission—remain largely missing [27,35]. To date, the only published survey from Mongolia that included molecular confirmation, conducted in the southern border province of Umnugobi, reported *E. canadensis* (G6/G7) infection 19.7% of camels and 6.7% of goats [28].

No comparable molecular data are available for the sheep, despite their large population size. Mongolia has ~ 24 million sheep and ongoing transmission, making it well placed to evaluate EG95 sheep vaccination as part of integrated control [36]. However, limited livestock genotype data hinders decision-making [37]. Globally, as molecular surveillance expands, reports of mixed-genotype transmission in CE-endemic countries are increasing, underscoring that without host-specific prevalence and genotype data, it is difficult to prioritize and tailor integrated control packages [38].

Our study aimed to address this evidence gap by assessing *E. granulosus s.l.* infection and genotype composition in key intermediate hosts in Mongolia, particularly sheep, to inform genotype-based, One Health control planning.

## Method

### Ethics statement

No live-animal experimentation or intervention was conducted. Liver and lung samples were collected post-mortem from animals slaughtered for commercial purposes, with no influence on the slaughter process. Formal ethical approval was not required, as the study involved post-mortem examination and did not involve procedures covered by institutional animal care protocols, in line with local ethical and veterinary guidelines. Written permission for sample collection was obtained from the slaughterhouse management.

### Sampling site

**Fig 1:**
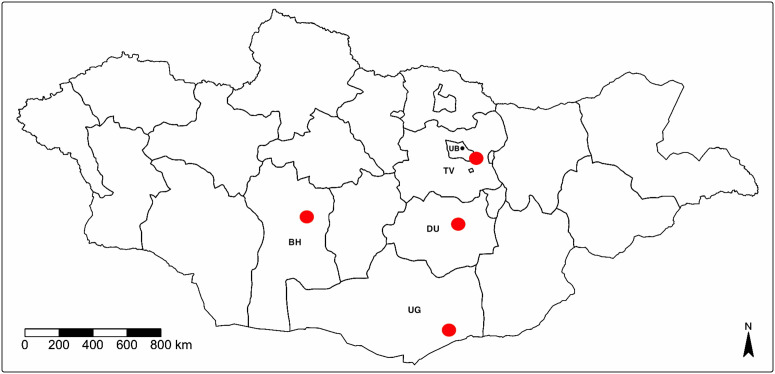
Study sampling sites. Red dots indicate sampling sites linked to each study province. Livestock from Bayankhongor (BH) and Dundgobi (DU) were sampled at abattoirs in the provincial centres (Bayankhongor town and Mandalgobi town, respectively). Livestock from Tuv province (TV) were sampled at the Nalaikh district abattoir in Ulaanbaatar city (UB, marked by the black dot), located near the administrative border between UB and TV and routinely slaughtering animals from TV. Samples from Umnugobi (UG) were collected at an open-air meat-processing site in Nomgon soum, near the Mongolia–China border. Administrative boundaries were obtained from the geoBoundaries database of political administrative boundaries [39]. Basemap source and download: https://www.geoboundaries.org/countryDownloads.html. geoBoundaries data are licensed under CC BY 4.0 with attribution required. Maps were created in R version 4.5.0.

We chose four provinces for our study: Bayankhongor (BH), Dundgobi (DU), Tuv (TV) and Umnugobi (UG) provinces to conduct a cross sectional, abattoir-based survey. Livestock from BH and DU were sampled at abattoirs in the provincial centres, Bayankhongor town (46°11′30″N, 100°43′04″E) and Mandalgobi soum – a smaller administrative unit of provinces (45°46′00″N, 106°16′11″E), respectively. Livestock from TV were sampled at the Nalaikh district abattoir in Ulaanbaatar (47°46′00″N, 107°16′59″E), located near the TV province border and routinely slaughters animals from TV. In UG, slaughtering was carried out at an abattoir facility in Nomgon soum, close to the southern border (42°09′51″N, 105°53′52″E) (Fig 1). These provinces represent distinct geographic regions and have reported higher numbers of human CE cases. They also share a nomadic pastoral lifestyle, large livestock herds, and free-roaming dogs that sustain *E. granulosus s.l.* transmission. We therefore selected them to increase the likelihood of capturing a broader diversity of genetic profiles. UG and BH represent the south, while DU and TV represent the central region among endemic areas. BH, DU, and TV each have large sheep populations (~1.2–2.1 million head), whereas UG has a much larger camel population, with ~0.10–0.15 million head comprising more than 30% of the country’s camels [34].

### Sample collection

From August to December 2024, we spent one week at each site and examined all animals slaughtered during that period as part of routine meat inspection. In all provinces except DU, the livers and lungs were inspected; in DU, only the livers were examined due to operational constraints at the abattoir. For each carcass, we recorded host species, origin (soum level = administrative unit below province level), affected organ(s), and the number of cysts. Cysts smaller than 1 cm (<1 cm) were not included in the analysis. Age was recorded inconsistently and thus excluded from the analyses; however, given that lambs and young animals are rarely slaughtered for food consumption in Mongolia, most examined animals were likely of middle to advanced age. Suspected cysts were aseptically excised, opened to assess cyst contents and characteristics, and placed into sterile 15–50 mL tubes; for very large cysts, a representative portion was collected. For each cyst, we recorded size and macroscopic characteristics (e.g., fluid-filled, calcified). Due to the remoteness of the sampling sites and limited access to microscopy facilities, the presence of protoscoleces was not systematically assessed. Samples were maintained in a cold chain (portable freezer with ice packs), stored at −20 °C, and transported to Ulaanbaatar for subsequent molecular analyses.

### Statistical analysis

Descriptive analyses used the total number of animals examined as the denominator for calculating percentage of animals with cystic lesions and the detection rate of *E. granulosus s.l.* infection. Each animal was counted as positive if at least one cyst was detected. Because sheep were the only species sampled in all provinces, provincial comparisons were restricted to sheep, while host-species comparisons used data from UG province, where multiple species were examined. Differences across provinces (sheep only) and among host species in UG were assessed using the Fisher–Freeman–Halton exact test. Where an overall difference was detected, pairwise Fisher exact tests with Holm adjustment were used to control for multiple comparisons. Exact 95% confidence intervals were reported for all estimates.

Macroscopic cyst characteristics (organ, size category, calcification and fluid content) were summarised using counts and percentages. We then conducted an exploratory analysis to assess whether cyst condition (calcification and presence of fluid) was associated with gene amplification success. Amplification rates were expressed as the proportion of cystic lesions positive for *E. granulosus s.l.* (n/N), with exact binomial 95% confidence intervals. Differences between categories were assessed using Fisher’s exact test. Cysts with missing data were excluded, and all tests were two-sided with a significance level of 0.05. Analyses were performed in R version 4.5.0 (R Foundation for Statistical Computing, Vienna, Austria).

### Molecular analysis

#### DNA extraction.

Cyst contents (including calcified tissue) and the germinal layer were first cut into small pieces with sterile scissors and then further mechanically homogenised using a handheld motorized tissue homogeniser. The host-derived fibrous capsule was not used. Genomic DNA was extracted using the QIAGEN DNeasy Blood & Tissue Kit (QIAGEN, Hilden, Germany) according to the manufacturer’s instructions. Proteinase K digestion at 56 °C was performed in two stages: an initial incubation for all samples, followed by an extended overnight incubation at 56 °C for samples containing firm or calcified material to ensure complete lysis. DNA was purified on silica columns, eluted in 50–100 µL of AE buffer, and stored at −20 °C until further analysis.

#### PCR amplification and sequencing.

After DNA extraction, all cyst DNA samples were tested for *E. granulosus s.l.* using mitochondrial *COX1* and *ATP6* II loci. Primer pairs were selected from the literature [40,41]. PCR conditions and reagents were optimised during assay setup across multiple runs using a subset of representative samples, and the final modified protocol, including locus-specific reaction mixtures and cycling conditions applied to the full dataset, is shown in S1 Table. Each PCR run included a positive control and a no-template control. Products were visualised on 2% agarose gel, and amplicons of the expected size were purified and Sanger sequenced bidirectionally using the amplification primers. Chromatograms were trimmed and assembled into consensus sequences.

#### Phylogenetic and haplotype analysis.

Mitochondrial *COX1* sequences obtained in this study, together with *E. granulosus s.l.* reference sequences from GenBank (including isolates from Mongolia, its neighboring countries - China, Russia and Kazakhtsan - and other endemic regions), were aligned using the MUSCLE algorithm implemented in BioEdit v5.0.9, and ambiguous positions were corrected manually. The final alignment was trimmed to a common length of 742 bp. To further distinguish *E. canadensis* lineages within the G6/G7 cluster, we compared our *COX1* sequences with the published positions reported by Laurimäe et al. (2019) that fall within our trimmed *COX1* alignment (742 bp). Based on these published diagnostic sites, *E. canadensis* sequences were interpreted as belonging to the G6, G7a or G7b genotype [42]. Species- and genotype-level relationships were inferred by constructing a phylogenetic tree in MEGA v11 using the maximum-likelihood (ML) method under a General Time Reversible model with gamma-distributed rate variation (GTR + Γ), and node support was evaluated with 1,000 bootstrap replicates. For rooting and external comparison, a *Versteria mustelae* (*V. mustelae*) mitochondrial sequence was included as an outgroup. *Taenia hydatigena* (*T. hydatigena)* and *Hydatigera kamiyai* were included as non-*Echinococcus* reference taxa. Trees were visualized and edited in MEGA v11 and annotated with genotype.

To explore genetic diversity and relationships among *E. granulosus s.l.* isolates, haplotypes were defined from the aligned mitochondrial *COX1* sequences. Pairwise genetic distances among *COX1* haplotypes were calculated in MEGA v11 using the Maximum Composite Likelihood approach. Genetic diversity indices, including the number of haplotypes (h), number of segregating sites (S), haplotype diversity (Hd), mean number of pairwise differences (K) and nucleotide diversity (π), were computed in DnaSP v6.12 for the overall dataset and selected subgroups. All defined haplotypes were checked by examining nucleotide base-quality scores at variable sites in FinchTV v1.4. To visualise fine-scale relationships among Mongolian isolates and to place them in a broader geographic and host context, we constructed a median-joining haplotype network in PopART v1.7, including reference sequences from other countries and host species. Maps were created in R version 4.5.0. Administrative boundaries were obtained from the geoBoundaries database of political administrative boundaries [39]. The geoBoundaries dataset is openly available under the CC BY 4.0 license.

## Results

### Data analysis

A total of 4,578 animals were examined, and the overall percentage of cystic lesions (without molecular confirmation) was 2.5% (115/4,578), yielding 140 cysts. Across all animals, *E. granulosus s.l.* was molecularly confirmed in 0.31% (14/4,578) of animals examined (Table 1). Most confirmed infections were *E. canadensis* (G6/G7 cluster) (13/14), detected in sheep from three sites (0.23%, 10/4,433), goats from UG (1.51%, 2/132), and one camel from UG (7.69%, 1/13). The remaining confirmed infection (1/14) was *E. granulosus s.s.* (G1/G3), identified in a sheep from TV (0.09%, 1/1,093). In our sample, *T. hydatigena* was also detected in 0.35% (16/4,578) of animals overall, with TV having the most infections (11/1,093), followed by BH (3/1,599), and DU (2/1,713).

**Table 1:** Distribution of cystic lesions and *E. granulosus s.l.* isolates by province and host species.

Province	Host	Animals examined (N)	Animals with cysts n/N (%)	Cysts collected (n)	Species	Isolates n/N (%)
**Bayankhongor (BH)**	Sheep	1599	21 (1.3)	27	*E. canadensis G6/G7*	2 (0.13)
**Dundgobi (DU)**	Sheep	1713	31 (1.8)	36	*–*	–
**Tuv (TV)**	Sheep	1093	50 (4.6)	58	*E. canadensis G6/G7*	7 (0.64)
					*E. granulosus s.l. G1/G3*	1 (0.09)
**Umnugobi (UG)**	Sheep	28	4 (14.3)	8	*E. canadensis G6/G7*	1 (3.57)
	Goat	132	6 (4.5)	8	*E. canadensis G6/G7*	2 (1.52)
	Camel	13	3 (23.1)	3	*E. canadensis G6/G7*	1 (7.69)
**Total**		**4578**	**115 (2.5)**	**140**	***E. granulosus s.l.***	**14 (0.31)**

*Denominator (N)- Number of animal examined.*

In sheep, the percentage of cystic lesions differed significantly by province (P < 0.001): 1.3% (21/1,599) in BH, 1.8% (31/1,713) in DU, 4.6% (50/1,093) in TV, and 14.3% (4/28) in UG. Among sheep, the percentage of confirmed G6/G7 was 0.13% (2/1,599) in BH, 0.64% (7/1,093) in TV, and 3.57% (1/28) in UG. Within UG, cystic lesions were detected in 4.5% (6/132) of goats and 23.1% (3/13) of camels, and these percentages differed significantly across host species (P = 0.02).

Organ localisation is reported only for cystic lesions (without molecular confirmation) from sites where both the liver and lungs were examined, comprising a total of 104 lesions. Among these, 82.7% (86/104) were located in the liver and 17.3% (18/104) in the lungs. Of the 14 PCR-confirmed *E. granulosus s.l.* cysts, 92.9% (13/14) were found in the liver and 7.1% (1/14) in the lung.

**Fig 2:**
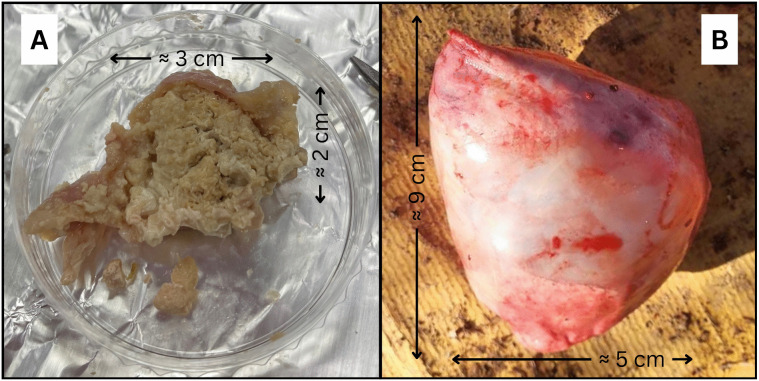
Macroscopic appearance of hydatid cysts collected from livestock. **(A)** Typical calcified cyst (opened to show contents), representing the majority of cysts in this study. Example from a sheep liver in Tuv province, ~ 3 cm diameter; **(B)** Rare fertile cyst containing clear fluid. Large (~5 x 9 cm), firm pulmonary cyst from a goat in Umnugobi province, molecularly confirmed as *E. canadensis* G6.

Cysts smaller than 1 cm (<1 cm) were not routinely collected. An exception was made for three <1 cm lung cysts from a single animal that also had a cyst larger than 1 cm (>1 cm) in the liver. These cysts were included in the overall cyst counts but excluded from the size analysis, leaving 137 cystic lesions available for size assessment. Among them, 62.8% (86/137) measured 1–3 cm, 12.4% (17/137) measured 4–5 cm, and 9.5% (13/137) were >5 cm; size was not recorded for 15.3% of cysts collected (21/137). Among molecularly confirmed *E. granulosus s.l.* cysts (n = 14), 78.6% (11/14) measured 1–3 cm, 7.1% (1/14) measured over 5 cm. Calcification was observed in 86.4% (121/140) of cystic lesions, whereas 9.3% (13/140) were not calcified; calcification status was missing for 4.3% (6/140). Visible fluid was present in 17.9% (25/140) of cystic lesions and absent in 75.7% (106/140); fluid status was missing for 6.4% (9/140). Among PCR-confirmed *E. granulosus s.l.* cysts (n = 14), 78.6% (11/14) were calcified and 28.6% (4/14) contained visible fluid. Macroscopic features of the cysts are shown in Fig 2, and a summary of the gross findings is presented in Table 2.

**Table 2:** Macroscopic characteristics of cystic lesions and cysts identified with *E. granulosus s.l.*

Feature	Category	*E. granulosus s.l.*, n/N (%) (N = 14)	95% CI
**Size**	1–3 cm	11 (78.6)	49.2–95.3
	4–5 cm	0 (0.0)	0.0–23.2
	>5 cm	1 (7.1)	0.2–33.9
	NA	2(14.3)	1.8–42.8
**Calcification**	Calcified	11 (78.6)	49.2–95.3
	Not calcified	2 (14.3)	1.8–42.8
	NA	1 (7.1)	0.2–33.9
**Fluid content**	Fluid present	4 (28.6)	8.4–58.1
	No fluid	10 (71.4)	41.9–91.6
	NA	0 (0.0)	0.0–23.2

Among the 121 calcified cysts, 11 (9.1%) yielded successful amplification, compared with 2 of 13 non-calcified cysts (15.4%); one of six cysts with missing calcification status was positive (16.7%). Regarding fluid content, amplification was obtained from 4 of 25 cysts with visible fluid (16.0%) and from 10 of 106 cysts without visible fluid (9.4%). Comparison of cysts with the “feature present” versus “feature absent” showed no statistically significant association between calcification and amplification success (P = 0.62) or between visible fluid and amplification success (P = 0.47) (S2 Table).

### Molecular analysis

#### Phylogenetic and haplotype analysis.

In total, we obtained mitochondrial gene sequences of *E. granulosus s.l.* from 14 cysts, each derived from a unique animal. These included nine partial *COX1* sequences (~800 bp) and five *ATP6* II sequences (600–700 bp). The nine *COX1* sequences were used to construct a phylogenetic tree (S1 Fig). In the tree, all Mongolian sequences clustered with reference sequences of either *E. canadensis* (G6/G7 cluster) or *E. granulosus s.s.* (G1/G3), with strong bootstrap support separating these two clades. Eight sequences were assigned to *E. canadensis* (G6/G7 cluster) and one to *E. granulosus s.s.* (G1/G3). *ATP6* II sequences were obtained from five cysts, and BLAST analysis showed that all five corresponded to *E. canadensis* G6/G7. All sequences have been deposited in GenBank (*COX1*: PX599341–PX599345; *ATP6* II: PX682355–PX682357). No cyst yielded sequences for both loci. Additionally, the ML tree confirmed the presence of *T.hydatigena*, with all six of the *COX1* sequences clustering with *T. hydatigena* reference sequences*.* As *T. hydatigena* was outside the scope of the present study, it was not included in further analysis. Metadata for the *T. hydatigena* isolates, including host, geographic origin, cyst characteristics, and phylogenetic tree, are provided in S3 Table and S1 Fig in the supplementary material.

**Fig 3:**
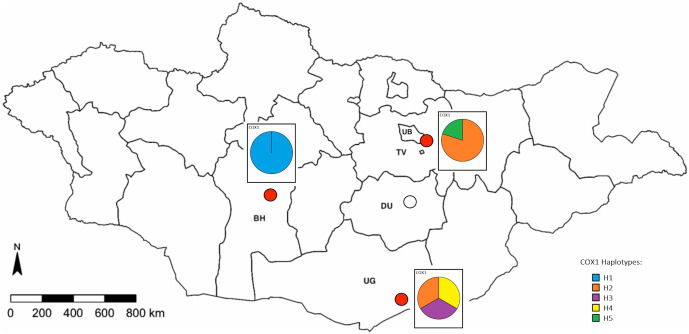
Geographic distribution of mitochondrial *COX1* haplotypes (742 bp) at the four sampling sites in Mongolia. BH – Bayankhongor province, DU – Dundgobi province, TV – Tuv province, UG – Umnugobi province, UB-Ulaanbaatar city. Administrative boundaries were obtained from the geoBoundaries database of political administrative boundaries [39]. Basemap source and download: https://www.geoboundaries.org/countryDownloads.html. geoBoundaries data are licensed under CC BY 4.0 with attribution required. Maps were created in R version 4.5.0.

In total, five *COX1* haplotypes (H1–H5) were identified among the 14 *E. granulosus s.l.* isolates. Analysis of the geographic distribution of *COX1* haplotypes showed that H2 was the only haplotype shared among multiple locations, occurring in both UG and TV. UG showed the highest haplotype diversity with three haplotypes (H2–H4) detected (Hd = 1.00). This was followed by TV, where two haplotypes (H2 and H5) were identified (Hd = 0.40). In contrast, only a single haplotype (H1) was detected in BH (Hd = 0.00), and no positive samples were obtained from DU. Maximum Composite Likelihood pairwise distances among our sequences ranged from 0.13% to 21.3% (0.001–0.213). Haplotypes H1–H4 formed a closely related G6/G7 cluster, differing from one another by 0.13–0.40% (0.001–0.004), consistent with a recently diversified lineage circulating across multiple hosts. Four haplotypes (H1–H4) belonged to *E. canadensis* G6/G7 cluster, with H1, H2, and H3 corresponding to the G6 genotype, and H4 corresponding to the G7a genotype. The remaining haplotype (H5) belonged to *E. granulosus s.s.* (G1/G3; Table 3) [42]. Similarly, three unique *ATP6 II* haplotypes (HA1–HA3) were identified among the five *E. canadensis* (G6/G7) sequences analysed. The geographic distribution showed clear site-specific structuring, with HA1 detected only in BH, HA2 restricted to UG, and HA3 found exclusively in TV, indicating no haplotype sharing among locations. No haplotype diversity was observed within any site (Hd = 0), as each location was represented by a single haplotype. Maximum Composite Likelihood pairwise distances among the five *ATP6II* sequences were low, ranging from 0.0–0.3% (0.0–0.003). Haplotypes HA2 and HA3 were the most closely related, showing a low divergence of 0.06% (0.0006), while HA1 showed moderate divergence from both HA2 and HA3, with pairwise distances of 0.24–0.3% (0.0024–0.003). Metadata for all 14 *E. granulosus s.l.* isolates (both *COX1* and *ATP6II*), including haplotype and genotype information, are presented in Table 3, while the geographic distribution of *COX1* haplotypes is shown in Fig 3.

**Table 3:** Metadata of *E. granulosus s.l.* cysts successfully genotyped from Mongolian livestock.

N	Province	Host	Organ	Cyst size (cm)	Cyst content	Gene sequenced	Hapl.	Accession no.	Genotype
1	BH	Sheep	Liver	1–3	Calcified	*COX1*	H1	PX599341	G6
2	UG	Goat	Liver	1–3	Calcified	*COX1*	H2	PX599342	G6
3	TV	Sheep	Liver	NA	Fluid*	*COX1*	H2	PX599342	G6
4	TV	Sheep	Liver	1–3	Calcified	*COX1*	H2	PX599342	G6
5	TV	Sheep	Liver	1–3	Calcified	*COX1*	H2	PX599342	G6
6	TV	Sheep	Liver	1–3	Calcified	*COX1*	H2	PX599342	G6
7	UG	Goat	Lung	>5	Fluid	*COX1*	H3	PX599343	G6
8	UG	Camel	Liver	1–3	Fluid	*COX1*	H4	PX599344	G7a
9	TV	Sheep	Liver	1–3	Fluid**	*COX1*	H5	PX599345	G1/G3
10	BH	Sheep	Liver	1–3	Calcified	*ATP6* II	HA1	PX682355	G6/G7
11	TV	Sheep	Liver	1–3	Calcified	*ATP6* II	HA 3	PX682357	G6/G7
12	TV	Sheep	Liver	1–3	Calcified	*ATP6* II	HA 3	PX682357	G6/G7
13	TV	Sheep	Liver	NA	Calcified	*ATP6* II	HA 3	PX682357	G6/G7
14	UG	Sheep	Liver	1–3	Calcified	*ATP6* II	HA 2	PX682356	G6/G7

*Calcification status was not available; **Fluid was present, with small calcification observed.

**Fig 4:**
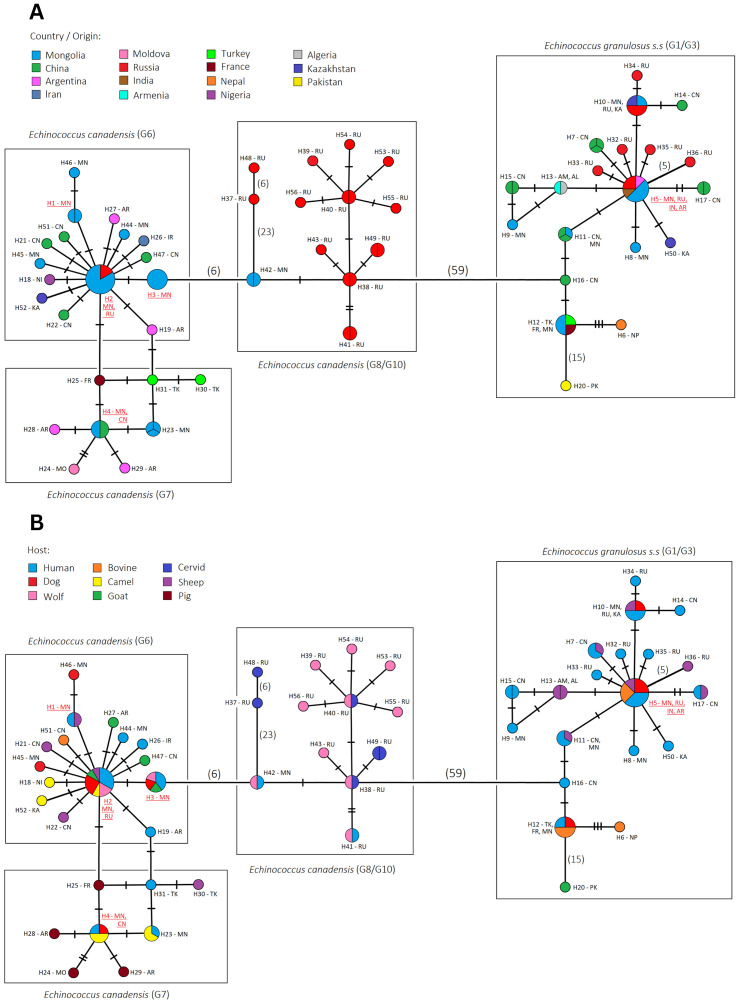
Median-Joining haplotype network of the *E. granulosus s.l.* complex using the mitochondrial *COX1* (742 bp) constructed in PopArt v1.7. Circles represent haplotypes, with size proportional to the number of individuals. Hatch marks show nucleotide substitutions between haplotypes (values >5 are shown numerically). **(A)** Haplotype sequences are coloured based on collection locations, and (B) haplotype sequences are coloured based on the host animal the parasite was collected from. Detailed information for all *COX1* sequences used in this study is provided in S4 and S5 Tables of the Supplementary material.

In the haplotype network, H2 was the most central and geographically widespread haplotype within the G6/G7 cluster. In our study it was detected in four sheep and one goat. Published data indicate that this haplotype has also been reported in humans, dogs, wolves, goats, and camels in Mongolia, as well as in humans and wolves in Russia. Haplotype H1 and H3 differed from H2 by a single nucleotide and were found in sheep and goats in our survey; both have previously been detected in human and dog infections in Mongolia. Haplotype H4 was slightly more divergent from H2, differing by three nucleotides, and was detected in a camel from UG. This haplotype corresponds to a lineage previously found in dog in Mongolia and in human cases in China. Several additional Mongolian G6/G7 haplotypes together with closely related sequences from China, Kazakhstan and other countries grouped with haplotype H1–H4. Outside this core G6/G7 group, haplotype H42 was the most divergent from H2, differing eight nucleotides. This haplotype has previously been reported from a wolf and a human in Mongolia and is one nucleotide away from a Russian haplotype found in wolves and cervids. The *COX1* haplotype network, together with reference sequences by country and host, is shown in Fig 4.

Haplotype H5 clustered within the *E. granulosus s.s.* (G1/G3). It was found in one sheep in our study and has previously been reported from humans and dogs in Mongolia, as well as from human infections in Russia and bovine hosts in India and Argentina (Fig 4). Multiple G1/G3 haplotypes (e.g., H7, H8, H10, H11, H13, H32, H33, H35, H36, H50) differ from H5 by a single nucleotide and have been recorded in humans and livestock across Mongolia, Russia, China, Kazakhstan and other countries (see S4 Table for sequence references). Mongolian host ranges of haplotypes H1–H5 are summarised in Table 4, and detailed sequence metadata and references for all *COX1* haplotypes included in the network are provided as supplementary in S4 and S5 Tables.

**Table 4:** Summary of previously reported and newly identified Mongolian host records for *COX1* haplotypes H1–H5.

Haplotype	Genotype	Previous reports from Mongolia			This survey
Human infection	Definitive host	Intermediate host (livestock)	Intermediate host (livestock)
**H1**	G6	Yes^a^	–	–	Sheep
**H2**	G6	Yes^a,^	Dog^c,d^, Wolf^b^	Goal^e,f^, Camel^e^	Goat, Sheep
**H3**	G6	Yes^a,c^	Dog^c^	–	Goat
**H4**	G7a	–	Dog^d^	–	Camel
**H5**	G1/G3	Yes^a^	Dog^c^	–	Sheep

**Note:** Superscript letters refer to the source records listed in S4 Table: a, Ito et al., 2014; b, Ito et al., 2013; c, GenBank/NCBI sequence submitted by Dorjsuren et al., 2018 (accession number provided in S4 Table); d, GenBank/NCBI sequence submitted by Tserendovdon et al., 2020 (accession number provided in S4 Table); e, Bold et al., 2019; and f, Biedermann et al., 2025.

## Discussion

In our study, *E. canadensis* (G6/G7) predominated among genotyped isolates (93%; 13/14), the majority of which were from sheep (11/14), whereas *E. granulosus s.s.* (G1/G3) was detected in one sheep (7%; 1/14). This distribution is consistent with genotyping evidence from Mongolia in humans and canids, as well as the published data from camels and goats, further supporting the predominance of G6/G7 in the country [28,32–34,43]. Haplotype analysis identified four closely related *COX1* G6/G7 haplotypes (H1–H4) forming a cluster in which H2 is central, while the single G1/G3 haplotype (H5) was detected in sheep. Our findings clarify transmission patterns in intermediate hosts and refine the current understanding of the CE genotype landscape in Mongolia, and providing evidence to support genotype-informed control strategies.

Geographically, *E. canadensis* (G6/G7) detection was highest in livestock from UG, the southernmost province bordering China, which also reports a high burden of human CE. UG has a relatively large camel population, accounting for roughly 30% of the country’s camels. Previous work from central UG reported abattoir infection in camels (19%) and goats (6%), with isolates confirmed as *E. canadensis* [28]. In our survey conducted closer to the border, infection rates were 7.7% in camels and 1.5% in goats, and we also detected *E. canadensis* in sheep (3.6%). This pattern may reflect a combination of ecological conditions, livestock composition, differences in the host associations of the circulating *Echinococcus* taxa, and local slaughterhouse practices. In particular, camels are often slaughtered in open field settings. Due to their size, offals are often discarded on site and can remain accessible to free-roaming dogs and wild canids over time, thereby increasing opportunities for transmission. Previous estimates have suggested an association between camel population size and human CE cases in Mongolia [28]. However, additional targeted studies that include fertility/viability assessment by host species are needed to clarify dominant transmission pathways and the relative contribution of sheep, goats and camels.

Notably, the camel-derived haplotype identified in UG (H4), assigned to the G7a genotype, matched the rare G7 haplotype reported from human CE cases detected in China (Heilongjiang and Guangdong) [44,45]. This is particularly important to note because human CE in China has historically been overwhelmingly attributed to G1/G3, whereas G6/G7 infections in humans remain exceptionally uncommon [46]. This shared haplotype represents a plausible transboundary signal. Although our data cannot determine transmission routes, the identical match raises a testable hypothesis that animal movement, trade pathways, or shared wildlife corridors may facilitate cross-border dispersal of G6/G7 lineages.

In TV, both *E. canadensis* (G6/G7) and *E. granulosus s.s.* (G1/G3) were detected. A previous study of human CE also reported both taxa from this area [32]. This central province is a vast steppe area with rich vegetation, which is suitable for sheep grazing, and it has around 2 million sheep (≈8% of Mongolia’s total). It has more frequent sheep–dog–human contact and could support the continued circulation of G1/G3. However, in our survey, G6/G7 was still much more frequent than G1/G3 in TV. To better interpret this mixed setting, it would be important to assess whether the viability/fertility of G6/G7 cysts in sheep is comparable to that of G1/G3. We have limited information from the rest of the country, particularly the western and northern regions. Therefore, nationwide genetic surveillance covering a larger geographic area and more host species could improve understanding of mixed-genotype dynamics and host interactions.

Of the five haplotypes identified in our study, the H2 haplotype, which was G6 genotype, is central to the G6/G7 cluster and has the broadest host and geographic range. Published data document the dominant H2 haplotype in wolves and humans on both the Mongolian and Russian sides of the Altai mountain region, suggesting that this lineage may circulate in wildlife (sylvatic) cycle through a shared transboundary ecosystem around the Altai region [34,47]. On the Mongolian side, H2 is also highly prevalent in domestic hosts, having been detected in camels, goats, sheep, dogs, and humans [28,32]. This suggests that H2 may link domestic and wildlife transmission in Mongolia, with both cycles potentially operating in parallel. In addition, H42, which is a more divergent haplotype than H2, was reported from a Mongolian human case and is closely related to lineages from the Russian wolf–cervid wildlife cycle [32,34,48].

The single G1/G3 haplotype (H5) was found in a sheep from TV. The H5 haplotype has previously been reported in Mongolian humans and dogs and shows close genetic similarity to G1/G3 haplotypes described across Eurasia and beyond, differing by only one nucleotide from multiple published sequences [32,43]. Although G6/G7 predominated in our livestock sample, detection of G1/G3 in sheep, dogs and humans suggests that both taxa are circulating in Mongolia.

Most cysts in our collection were small (63%) and calcified (86%). Molecularly confirmed *E. canadensis* G6/G7 cysts in our study were likewise predominantly small and heavily calcified (Table 3). This pattern may have several explanations. First, it may reflect intrinsic differences between genotype groups: studies have reported genotype-linked differences in cyst phenotype, with G6/G7 more often presenting as smaller cysts compared with G1/G3 in both humans and livestock [49–52]. Second, host–parasite compatibility may contribute: reports suggest that G1/G3 is more often fertile in sheep than in camels or pigs, whereas G6/G7 detected in small ruminants is more frequently calcified or non-fertile, with higher fertility more commonly reported in camels [53,54]. In less-preferred hosts, cyst development may be attenuated, favoring degeneration and calcification. This may be reflected in our samples from sheep, which suggest reduced cyst viability in sheep in the surveyed settings; however, this requires further confirmation and assessment of cyst fertility/viability. In addition, stronger immune status in Mongolian sheep in traditional pastoral settings could also contribute to cyst inactivation and degeneration [55]. Importantly, small, fast-degenerating (often calcified) cysts can have implications for the clinical presentation and prognosis of CE in humans. Mixed-genotype settings such as Mongolia can be valuable for studying phenotypical differences.

The amplification rate prior to sequencing was low, and overall genotyping success was 10% (14/140). This likely reflects a combination of factors, including the high proportion of degenerated or calcified lesions, which may contain limited parasite tissue and PCR inhibitors, as well as lesion heterogeneity [56–58]. Not all gross cystic lesions represented *E. granulosus s.l.*, as supported by the identification of *T. hydatigena* in a subset We also observed slightly higher amplification in non-calcified cysts and cysts with visible fluid (S2 Table), but these differences were not statistically significant, although the small numbers limit the power of this comparison. Overall, PCR amplification from abattoir-derived lesions collected in remote field settings can be challenging, particularly for heavily calcified material. Future surveys in similar settings may therefore require adapted sampling and DNA extraction approaches to maximise DNA yield, as well as amplification and sequencing success [17].

In total, cystic lesions were detected in 2.5% of examined animals. Of these infections 12% (14/115) were *E. granulosus s.l.* and 14% (16/115) were *T. hydatigena.* This means that the overall prevalence of cystic lesions cannot be equated with that of CE. Co-occurrence of the two cestodes is not unexpected, given that both parasites share dogs/wild canids as definitive hosts and sheep/goats as intermediate hosts, so they frequently co-circulate on the same farms/abattoirs [59,60]. The WOAH manual explicitly warns of the possible miscalculation of CE estimates in animals where the *T. hydatigena* is also common, and stressed the importance of PCR confirmation [17,61]. It is worth mentioning that in some control or surveillance settings, *T. hydatigena* lesions have been used as a practical proxy indicator of taeniid transmission pressure in livestock [9,62,63].

A key implication of our study is that the genotype distribution observed in sheep raises important questions about the effectiveness of EG95 vaccination in Mongolia. The EG95 vaccine is highly effective in *E. granulosus s.l.* (G1/G3) dominated regions. However evidence of cross-protection against *E. canadensis* (G6/G7) remains limited and inconsistent [12,18]. In Mongolia, despite having no surveillance for CE, overall, accumulated molecular studies consistently report a higher proportion of *E. canadensis* in both human and animal infections [28,32–35,43]. Vaccine impact may therefore be lower than in settings where G1/G3 is more prevalent [9,18]. However, broader One Health surveys across additional provinces and host species are needed to confirm the national picture, and genotype distribution should inform decision-making. As genotype mapping expands, more endemic countries may be recognised as mixed-genotype settings, where a uniform control package might be less optimal [38]. For instance, where G1/G3 predominates, combining dog deworming with targeted sheep vaccination may be appropriate, whereas in G6/G7-dominated settings greater impact may come from prioritising dog deworming alongside strengthened slaughterhouse/offal management and other measures.

Another important implication is the potential for cross-border transmission of *E. canadensis* lineages, especially into neighbouring China. Ongoing EG95 vaccination programmes in sheep in China have been highly successful, achieving substantial reductions in prevalence and transmission [16,64]. Increased circulation of non-vaccine-matched genotypes could undermine these gains [46]. This risk is heightened by the rapidly growing trade in live animals and animal products between Mongolia and China: In 2023 alone, Mongolia exported approximately 80 thousand tonnes of meat, with China remaining a major destination, and since 2022 more than 20 thousand live livestock, mainly sheep, have been exported to China, which may increase in the coming years [65]. More broadly, uncontrolled transnational spread of non-vaccine-matched genotypes poses an emerging challenge to regional control and elimination efforts. A jointly coordinated assessment of cross-border transmission risk between the two countries is needed to address this issue [66].

This study has several limitations. First, during the sample collection, systematic microscopic or histopathological examination for presence of protoscoleces was not performed due to the remote setting, and cyst fertility and viability were not assessed. Therefore, our data are largely limited in comparing the relative contribution of sheep, goats, and camels to maintaining transmission. However, the high proportion of calcified lesions suggests that many cysts were likely inactive, which may partially mitigate the lack of fertility assessment. Second, sampling was conducted under routine slaughterhouse conditions, where the rapid pace of processing prevented consistent recording of animal age and restricted organ examination to the liver and lungs. Age-related heterogeneity may have been limited because slaughtered animals are typically adult in Mongolia, while lambs and very young animals are less commonly sent for meat production. Third, the high proportion of calcified cysts likely reduced DNA yield and PCR success rates, which may have led to an underestimation of true *E. granulosus s.l.* prevalence. Given these limitations, infection and prevalence estimates should be interpreted with caution. Finally, our genotyped isolates were heavily skewed towards sheep; this was intentional because sheep are the primary target species for vaccination, but the findings may not fully generalise to other livestock.

Our study provides further molecular evidence that *E. granulosus s.l.* infections in Mongolian livestock are largely associated with *E. canadensis* G6/G7, consistent with previous reports in camels, goats, humans and canids. The relatively high prevalence observed in camels in the southern province surveyed suggests that their role in transmission warrants further investigation. For the first time, we also show that *E. canadensis* G6/G7 predominated among the successfully genotyped sheep isolates in the surveyed areas. These findings provide evidence to inform future control strategies, particularly in mixed-genotype settings, by emphasising non-vaccine measures such as dog deworming and safe offal disposal, while also supporting careful vaccination planning. Given the limited surveillance system in Mongolia, strengthening it and establishing baseline data will be important for monitoring transmission patterns over time. By linking major human- and canine-derived haplotypes to specific livestock intermediate hosts, and by revealing close genetic connections with isolates from neighbouring countries, our results improve understanding of CE transmission dynamics in Mongolia and the wider region. This regional links underscore the need for coordinated cross-border surveillance and control efforts.

## Supporting information

S1 Table PCR primers and cycling conditions used for amplification of mitochondrial loci from cyst DNA. (DOCX)

S2 Table Successful amplification rate of *E. granulosus s.l.* by cyst condition. (DOCX)

S1 Fig Phylogenetic tree of *E. granulosus s.l.* and *T. hydatigena* based on the mitochondrial *COX1* gene. (TIF)

S3 Table Metadata *for T. hydatigena* identified. (DOCX)

S4 Table Metadata and genotype assignments for reference *COX1* haplotypes included in the network analysis. (DOCX)

S5 Table Haplotype frequency for countries and hosts. (DOCX)

S1 Dataset. Dataset used for the descriptive analyses of cyst characteristics.
(XLSX)
