# Supplementary material for: Molecular evidence of Echinococcus canadensis (G6/G7) predominance in Mongolian livestock and its implications for control
Source: PLoS Negl Trop Dis. 2026 Jun 15;20(6):e0014433. doi: 10.1371/journal.pntd.0014433 (PMC13278582; doi:10.1371/journal.pntd.0014433)
Supplement: S1 Table — (DOCX) [file pntd.0014433.s001.docx]

**S1.Table PCR primers and cycling conditions used for amplification of mitochondrial loci from cyst DNA.**

| **Locus** | **Primer** | **Sequence (5′–3′)** | **PCR reaction mix (25 µL)** | **Cycling conditions** |
| --- | --- | --- | --- | --- |
| *COX1* | Forward | TTGAATTTGCCACGTTTGAATGC | 12.5 µL 2× Blue Taq PCR Master Mix; 0.5 µL each primer (10 µM); 1 µL template DNA; nuclease-free water to 25 µL | 96 °C 2 min; 34 cycles of 95 °C 30 s, 50 °C 45 s, 72 °C 90 s; final extension 72 °C 10 min |
| *COX1* | Reverse | GAACCTAACGACATAACATAATGA | as above | as above |
| *ATP6* II | Forward | AAACTGTAGGGTTCATGTC | as above | 94 °C 2 min; 35 cycles of 94 °C 30 s, 54 °C 30 s, 72 °C 60 s; final extension 72 °C 5 min |
| *ATP6* II | Reverse | CAAAACCCGAATAATCTATC | as above | as above |
