# Supplementary material for: Molecular evidence of Echinococcus canadensis (G6/G7) predominance in Mongolian livestock and its implications for control
Source: PLoS Negl Trop Dis. 2026 Jun 15;20(6):e0014433. doi: 10.1371/journal.pntd.0014433 (PMC13278582; doi:10.1371/journal.pntd.0014433)
Supplement: S2 Table — (DOCX) [file pntd.0014433.s002.docx]

**S2 Table Successful amplification rate of E. granulosus s.l. by cyst condition**

| **Feature** | **Category** | ***E. granulosus* s.l. n/N (%)** | **95% CI** | **p-value*** |
| --- | --- | --- | --- | --- |
| **Calcification**  **N=140** | Calcified | 11/121 (9.1) | 4.6 – 15.7 | 0.62 |
|  | Not calcified | 2/13 (15.4) | 4.3–42.2 |  |
|  | NA | 1/6 (16.7) | 3.0–56.4 |  |
| **Fluid content**  **N=140** | Fluid present | 4/25 (16.0) | 6.4–34.7 | 0.47 |
|  | No fluid | 10/106 (9.4) | 4.6 – 16.5 |  |
|  | NA | 0/9 (0.0) | 0.0–29.9 |  |

**p-values from Fisher’s exact test comparing “feature present” vs “feature absent,” excluding cysts with missing data (NA).*
