# Supplementary figures and images for: Molecular evidence of Echinococcus canadensis (G6/G7) predominance in Mongolian livestock and its implications for control
Source: PLoS Negl Trop Dis. 2026 Jun 15;20(6):e0014433. doi: 10.1371/journal.pntd.0014433 (PMC13278582; doi:10.1371/journal.pntd.0014433)

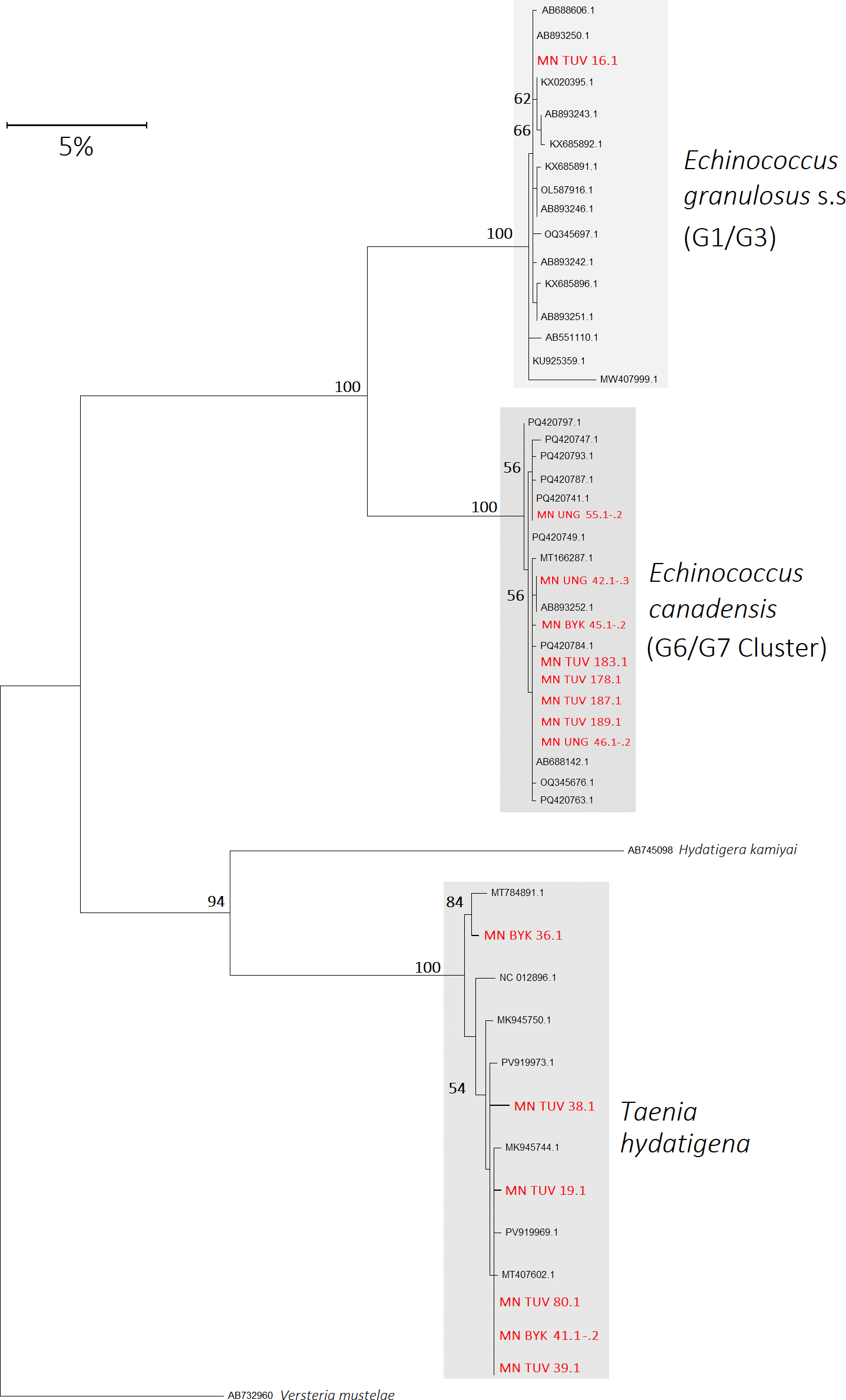

Supplement: S1 Fig — (TIF) [file pntd.0014433.s003.tif]
