# Supplementary material for: Molecular evidence of Echinococcus canadensis (G6/G7) predominance in Mongolian livestock and its implications for control
Source: PLoS Negl Trop Dis. 2026 Jun 15;20(6):e0014433. doi: 10.1371/journal.pntd.0014433 (PMC13278582; doi:10.1371/journal.pntd.0014433)
Supplement: S3 Table — (DOCX) [file pntd.0014433.s004.docx]

S3 Table. Meta data for *Taenia hydatigena* identified

| N | Province | Host | Organ | Cyst size (cm) | Cyst content | Gene sequenced | Label in phylogenetic tree (Fig.S1) |
| --- | --- | --- | --- | --- | --- | --- | --- |
| 1 | Tuv | Sheep | Liver | >5 | Fluid | *COX1, ATP6 II* | MN_Tuv 80.1 |
| 2 | Tuv | Sheep | Liver | 1–3 | Calcified | *COX1, ATP II* | MN_Tuv 19.1 |
| 3 | BH | Sheep | Liver | 1–3 | Calcified/small liquid | *COX1, ATP II* | MN_BYK 36.1 |
| 4 | Tuv | Sheep | Liver | 1–3 | Calcified | *COX1, ATP II* | MN_Tuv 38.1 |
| 5 | Tuv | Sheep | Liver | 3-5 | Calcified | *COX1, ATP II* | MN_Tuv 39.1 |
| 6 | BH | Sheep | Liver | 1–3 | Calcified | *COX1, ATP II* | MN_BYK 41.1 |
| 7 | BH | Sheep | Liver | 3-5 | Calcified/small liquid | *ATP6 II* | - |
| 8 | DU | Sheep | Liver | >5 | Calcified | *ATP6 II* | - |
| 9 | Tuv | Sheep | Liver | 1–3 | Calcified | *ATP6 II* | - |
| 10 | Tuv | Sheep | Liver | NA | NA | *ATP6 II* | - |
| 11 | Tuv | Sheep | Liver | 3-5 | Calcified | *ATP6 II* | - |
| 12 | Tuv | Sheep | Liver | 1-3 | Calcified | *ATP6 II* | - |
| 13 | Tuv | Sheep | Liver | 1-3 | Calcified | *ATP6 II* | - |
| 14 | DU | Sheep | Liver | >5 | Small liquid | *ATP6 II* | - |
| 15 | Tuv | Sheep | Liver | 1-3 | Calcified | *ATP6 II* | - |
| 16 | Tuv | Sheep | Liver | 1-3 | Calcified | *ATP6 II* | - |
