# Supplementary material for: Molecular evidence of Echinococcus canadensis (G6/G7) predominance in Mongolian livestock and its implications for control
Source: PLoS Negl Trop Dis. 2026 Jun 15;20(6):e0014433. doi: 10.1371/journal.pntd.0014433 (PMC13278582; doi:10.1371/journal.pntd.0014433)
Supplement: S4 Table — (DOCX) [file pntd.0014433.s005.docx]

S4 Table. Metadata and genotype assignments for reference *COX1* haplotypes included in the network analysis.

| **Haplotype** | **Country** | **Host** | **Accession** | **Reference at NCBI** | **Reported Genotype (bp)** | **Refined Genotype* (bp)** |
| --- | --- | --- | --- | --- | --- | --- |
| H1 | Mongolia | **Sheep** | PX599341 | This Study | G6 (13k) | G6 (13k) |
| H2 | Mongolia | **Sheep** | PX599342 | This Study | G6 (13k) | G6 (13k) |
| H3 | Mongolia | **Goat** | PX599343 | This Study | G6 (13k) | G6 (13k) |
| H4 | Mongolia | **Camel** | PX599344 | This Study | G7a (13k) | G7 (13k) |
| H5 | Mongolia | **Sheep** | PX599345 | This Study | G1/G3 (0.8k) | G1 (13k) |
| H6 | Nepal | Buffalo | AB551110.1 | [1] | G1 (1.6K) | G1 (1.6k) |
| H5 | Russia | Human | AB688136.1 | [2] | G1 (1.6K) | G1 (13k) |
| H32 | Russia | Human | AB688137.1 |  | G1 (1.6K) | G1 (1.6k) |
| H33 | Russia | Human | AB688138.1 |  | G1 (1.6K) | G1 (1.6k) |
| H10 | Russia | Human | AB688139.1 |  | G1 (1.6K) | G1 (1.6k) |
| H34 | Russia | Human | AB688140.1 |  | G1 (1.6K) | G1 (1.6k) |
| H35 | Russia | Human | AB688141.1 |  | G1 (1.6K) | G1 (1.6k) |
| H2 | Russia | Human | AB688142.1 |  | G6 (1.6K) | G6 (13k) |
| H7 | China | Human | AB688606.1 | [3] | G1 (1.6K) | G1 (1.6k) |
| H36 | Russia | Sheep | AB777905.1 | [4] | G1 (1.6K) | G1 (1.6k) |
| H10 | Russia | Sheep | AB777906.1 |  | G1 (1.6K) | G1 (1.6k) |
| H5 | Russia | Human | AB777908.1 |  | G1 (1.6K) | G1 (13k) |
| H2 | Russia | Wolf | AB777909.1 |  | G6 (1.6K) | G6 (13k) |
| H37 | Russia | Moose | AB777910.1 |  | G8 (1.6K) | G8 (1.6k) |
| H38 | Russia | Moose | AB777911.1 |  | G10 (1.6K) | G10 (1.6k) |
| H39 | Russia | Wolf | AB777912.1 |  | G10 (1.6K) | G10 (1.6k) |
| H40 | Russia | Reindeer | AB777913.1 |  | G10 (1.6K) | G10 |
| H41 | Russia | Human | AB777914.1 |  | G10 (1.6K) | G10 (1.6k) |
| H2 | Mongolia | Wolf | AB813183.1 | [5] | G6/G7 (1.6K) | G6 (13k) |
| H42 | Mongolia | Wolf | AB813184.1 |  | G10 (1.6K) | G10 (1.6k) |
| H8 | Mongolia | Human | AB893242.1 | [6] | G1 (1.6K) | G1 (1.6k) |
| H9 | Mongolia | Human | AB893243.1 |  | G1 (1.6K) | G1 (1.6k) |
| H10 | Mongolia | Human | AB893246.1 |  | G1 (1.6K) | G1 (1.6k) |
| H5 | Mongolia | Human | AB893250.1 |  | G1 (1.6K) | G1 (13k) |
| H11 | Mongolia | Human | AB893251.1 |  | G1 (1.6K) | G1 (1.6k) |
| H3 | Mongolia | Human | AB893252.1 |  | G6/G7 (1.6K) | G6 (13k) |
| H2 | Mongolia | Human | AB893259.1 |  | G6/G7 (1.6K) | G6 (13k) |
| H44 | Mongolia | Human | AB893261.1 |  | G6/G7 (1.6K) | G6 (1.6k) |
| H1 | Mongolia | Human | AB893262.1 |  | G6/G7 (1.6K) | G6 (13k) |
| H23 | Mongolia | Human | AB893263.1 |  | G6/G7 (1.6K) | G7 (13k) |
| H42 | Mongolia | Human | AB893264.1 |  | G10 (1.6K) | G10 (1.6k) |
| H47 | China | Goat | JQ317990.1 | [7] | G7 (0.8k) | G7 (0.8k) |
| H4 | China | Human | KJ556997.1 | [8] | G7 (0.8k) | G7 (13k) |
| H10 | Kazakhstan | Dog | KT001396.1 | [9] | G1/G3 (0.8k) | G1 (1.6k) |
| H12 | Turkey | Cattle | KU925359.1 | [10] | G1 (9k) | G1 (13k) |
| H13 | Armenia | Sheep | KX020395.1 | Sequence submitted by Ebi et al. 2016 | G1/G3 (1.6K) | G1/G3 |
| H11 | China | Human | KX685890.1 | [11] | G1/G3 (0.8k) | G1 (1.6k) |
| H14 | China | Human | KX685891.1 |  | G1/G3 (0.8k) | G1/G3 |
| H15 | China | Human | KX685892.1 |  | G1/G3 (0.8k) | G1/G3 |
| H7 | China | Human | KX685893.1 |  | G1/G3 (0.8k) | G1/G3 (0.8k) |
| H16 | China | Human | KX685896.1 |  | G1/G3 (0.8k) | G1/G3 |
| H17 | China | Human | KX685897.1 |  | G1/G3 (0.8k) | G1/G3 |
| H12 | France | Cattle | KY766889.1 | [12] | G1 (13k) | G1 (13k) |
| H5 | India | Buffalo | KY766891.1 |  | G1 (13k) | G1 (13k) |
| H48 | Russia | Elk | LC184604.1 | [13] | G8 (1.6K) | G8 (1.6k) |
| H49 | Russia | Elk | LC184605.1 |  | G10 (1.6K) | G10 (13k) |
| H2 | Mongolia | Human | LC384014.1 | Sequence submitted by Dorjsuren et al. 2018 | G6/G7 (0.8k) | G6 (13k) |
| H2 | Mongolia | Human | LC469709.1 |  | G6/G7 (0.8k) | G6 (13k) |
| H5 | Argentina | Cattle | MG672207.1 | [14] | G1 (13k) | G1 (13k) |
| H50 | Kazakhstan | Human | MG672257.1 |  | G1 (13k) | G1 (13k) |
| H13 | Algeria | Sheep | MG808340.1 | [15] | G1/G3 (1.6K) | G1/G3 |
| H2 | Mongolia | Camel | MK321258.1 | [16] | G6/G7 (1.6K) | G6 (13k) |
| H23 | Mongolia | Camel | MK321260.1 |  | G6/G7 (1.6K) | G7 (13k) |
| H2 | Mongolia | Dog | MK370102.1 | Sequence submitted by Dorjsuren et al. 2019 | G6/G7 (0.8k) | G6 (13k) |
| H3 | Mongolia | Dog | MK370105.1 |  | G6/G7 (0.8k) | G6 (13k) |
| H3 | Mongolia | Human | MK370106.1 |  | G6/G7 (0.8k) | G6 (13k) |
| H3 | Mongolia | Dog | MK370107.1 |  | G6/G7 (0.8k) | G6 (13k) |
| H5 | Mongolia | Dog | MK370109.1 |  | G1 (0.8k) | G1 (13k) |
| H5 | Mongolia | Dog | MK370110.1 |  | G1 (0.8k) | G1 (13k) |
| H12 | Mongolia | Human | MK370112.1 |  | G1/G3 (0.8k) | G1 (13k) |
| H12 | Mongolia | Dog | MK370113.1 |  | G1 (0.8k) | G1 (13k) |
| H51 | China | Yak | MN340038.1 | [17] | G6 (13k) | G6 (13k) |
| H18 | Nigeria | Camel | MT166287.1 | [18] | G6 (13k) | G6 (13k) |
| H19 | Argentina | Human | MT800784.1 | [19] | G6/G7 (1.6K) | G6 (1.6k) |
| H2 | Mongolia | Dog | MW084672.2 | Sequence submitted by Tserendovdon  et al. 2020 | G6/G7 (0.8k) | G6 (13k) |
| H45 | Mongolia | Dog | MW084676.2 |  | G6/G7 (0.8k) | G6/G7 (0.8k) |
| H46 | Mongolia | Dog | MW084694.2 |  | G6/G7 (0.8k) | G6/G7 (0.8k) |
| H4 | Mongolia | Dog | MW084699.2 |  | G6/G7 (0.8k) | G7 (13k) |
| H2 | Mongolia | Dog | MW084952.2 |  | G6/G7 (0.8k) | G6 (13k) |
| H20 | Pakistan | Goat | MW407999.1 | Sequence submitted by Simsek et al. 2020 | G1/G3 (0.8k) | G1/G3 |
| H52 | Kazakhstan | Camel | NC_011121.1 | [20] | G6 (13k) | G6 (13k) |
| H15 | China | Human | OP413398.1 | Sequence submitted by Wang et al 2022 | G1/G3 (0.8k) | G1/G3 |
| H49 | Russia | Moose | OQ161121.1 | [21] | G10 (13k) | G10 (13k) |
| H21 | China | Sheep | OQ345675.1 | [22] | G1/G3 (1.6K) | G1/G3 |
| H22 | China | Sheep | OQ345676.1 |  | G1/G3 (1.6K) | G1/G3 |
| H17 | China | Sheep | OQ345697.1 |  | G1/G3 (1.6K) | G1/G3 |
| H7 | China | Sheep | OQ345718.1 |  | G1/G3 (1.6K) | G1/G3 (1.6k) |
| H11 | China | Sheep | OQ345757.1 |  | G1/G3 (1.6K) | G1 (1.6k) |
| H41 | Russia | Wolf | OR420691.1 | [23] | G10 (1.6K) | G10 (1.6k) |
| H53 | Russia | Wolf | OR420693.1 |  | G10 (1.6K) | G10 (1.6k) |
| H54 | Russia | Wolf | OR420694.1 |  | G10 (1.6K) | G10 (1.6k) |
| H55 | Russia | Wolf | OR420696.1 |  | G10 (1.6K) | G10 (1.6k) |
| H56 | Russia | Wolf | OR420697.1 |  | G10 (1.6K) | G10 (1.6k) |
| H40 | Russia | Wolf | OR420699.1 |  | G10 (1.6K) | G10 |
| H57 | Russia | Wolf | OR420701.1 |  | G10 (1.6K) | G10 (1.6k) |
| H38 | Russia | Wolf | OR420702.1 |  | G10 (1.6K) | G10 (1.6k) |
| H4 | China | Human | PP158976.1 | [24] | G7 (0.8k) | G7 (13k) |
| H23 | Mongolia | Camel | PQ420741.1 | [25] | G7 (13k) | G7 (13k) |
| H2 | Mongolia | Goat | PQ420746.1 |  | G6 (13k) | G6 (13k) |
| H24 | Moldova | Pig | PQ420747.1 |  | G7 (13k) | G7 (13k) |
| H25 | France | Pig | PQ420749.1 |  | G7 (13k) | G7 (13k) |
| H26 | Iran | Human | PQ420763.1 |  | G6 (13k) | G6 (13k) |
| H27 | Argentina | Goat | PQ420784.1 |  | G6 (13k) | G6 (13k) |
| H28 | Argentina | Pig | PQ420787.1 |  | G7 (13k) | G7 (13k) |
| H29 | Argentina | Pig | PQ420793.1 |  | G7 (13k) | G7 (13k) |
| H30 | Turkey | Sheep | PQ420797.1 |  | G7 (13k) | G7 (13k) |
| H31 | Turkey | Human | PQ420798.1 |  | G7 (13k) | G7 (13k) |

** - Refined genotype assignment inferred from comparison with longer COX1 sequences from other studies included in the network analysis and from diagnostic positions reported by Laurimäe et al. (2019* [26]*).*

**References:**

1. Joshi DD, Joshi AB, Joshi H. Epidemiology of echinococcosis in Nepal. The Southeast Asian Journal of Tropical Medicine and Public Health. 1997 Jan 1;28:26-31.
2. Konyaev SV, Yanagida T, Ingovatova GM, Shoikhet YN, Nakao M, Sako Y, Bondarev AY, Ito A. Molecular identification of human echinococcosis in the Altai region of Russia. Parasitology International. 2012 Dec 1;61(4):711-4.
3. Yanagida T, Mohammadzadeh T, Kamhawi S, Nakao M, Sadjjadi SM, Hijjawi N, Abdel-Hafez SK, Sako Y, Okamoto M, Ito A. Genetic polymorphisms of Echinococcus granulosus sensu stricto in the Middle East. Parasitology International. 2012 Dec 1;61(4):599-603.
4. Konyaev SV, Yanagida T, Nakao M, Ingovatova GM, Shoykhet YN, Bondarev AY, Odnokurtsev VA, Loskutova KS, Lukmanova GI, Dokuchaev NE, Spiridonov S. Genetic diversity of Echinococcus spp. in Russia. Parasitology. 2013 Nov;140(13):1637-47.
5. Ito A, Chuluunbaatar G, Yanagida T, Davaasuren A, Sumiya B, Asakawa M, Ki T, Nakaya K, Davaajav A, Dorjsuren T, Nakao M. Echinococcus species from red foxes, corsac foxes, and wolves in Mongolia. Parasitology. 2013 Nov;140(13):1648-54.
6. Ito A, Dorjsuren T, Davaasuren A, Yanagida T, Sako Y, Nakaya K, Nakao M, Bat-Ochir OE, Ayushkhuu T, Bazarragchaa N, Gonchigsengee N. Cystic echinococcoses in Mongolia: molecular identification, serology and risk factors. PLoS neglected tropical diseases. 2014 Jun 19;8(6):e2937.
7. Ma J, Wang H, Lin G, Craig PS, Ito A, Cai Z, Zhang T, Han X, Ma X, Zhang J, Liu Y. Molecular identification of Echinococcus species from eastern and southern Qinghai, China, based on the mitochondrial cox1 gene. Parasitology Research. 2012 Jul;111(1):179-84.
8. Zhang T, Yang D, Zeng Z, Zhao W, Liu A, Piao D, Jiang T, Cao J, Shen Y, Liu H, Zhang W. Genetic characterization of human-derived hydatid cysts of Echinococcus granulosus sensu lato in Heilongjiang Province and the first report of G7 genotype of E. canadensis in humans in China. PloS one. 2014 Oct 16;9(10):e109059.
9. Boufana B, Lett W, Lahmar S, Griffiths A, Jenkins DJ, Buishi I, Engliez SA, Alrefadi MA, Eljaki AA, Elmestiri FM, Reyes MM. Canine echinococcosis: genetic diversity of Echinococcus granulosus sensu stricto (ss) from definitive hosts. Journal of helminthology. 2015 Nov;89(6):689-98.
10. Kinkar L, Laurimäe T, Simsek S, Balkaya I, Casulli A, Manfredi MT, Ponce-Gordo F, Varcasia A, Lavikainen A, Gonzalez LM, Rehbein S. High-resolution phylogeography of zoonotic tapeworm Echinococcus granulosus sensu stricto genotype G1 with an emphasis on its distribution in Turkey, Italy and Spain. Parasitology. 2016 Nov;143(13):1790-801.
11. Shang J, Zhang G, Yu W, He W, Wang Q, Zhong B, Wang Q, Liao S, Chen F, Huang Y. Molecular characterization of human echinococcosis in Sichuan, Western China. Acta tropica. 2019 Feb 1;190:45-51.
12. Kinkar L, Laurimäe T, Sharbatkhori M, Mirhendi H, Kia EB, Ponce-Gordo F, Andresiuk V, Simsek S, Lavikainen A, Irshadullah M, Umhang G. New mitogenome and nuclear evidence on the phylogeny and taxonomy of the highly zoonotic tapeworm Echinococcus granulosus sensu stricto. Infection, Genetics and Evolution. 2017 Aug 1;52:52-8.
13. Yanagida T, Lavikainen A, Hoberg EP, Konyaev S, Ito A, Sato MO, Zaikov VA, Beckmen K, Nakao M. Specific status of Echinococcus canadensis (Cestoda: Taeniidae) inferred from nuclear and mitochondrial gene sequences. International journal for parasitology. 2017 Dec 1;47(14):971-9.
14. Kinkar L, Laurimäe T, Acosta-Jamett G, Andresiuk V, Balkaya I, Casulli A, Gasser RB, van Der Giessen J, González LM, Haag KL, Zait H. Global phylogeography and genetic diversity of the zoonotic tapeworm Echinococcus granulosus sensu stricto genotype G1. International Journal for Parasitology. 2018 Aug 1;48(9-10):729-42.
15. Laatamna AE, Ebi D, Brahimi K, Bediaf K, Wassermann M, Souttou K, Romig T. Frequency and genetic diversity of Echinococcus granulosus sensu stricto in sheep and cattle from the steppe region of Djelfa, Algeria. Parasitology Research. 2019 Jan 23;118(1):89-96.
16. Bold B, Boué F, Schindler C, Badmaa B, Batbekh B, Argamjav B, Bayasgalan C, Ito A, Narankhuu U, Shagj A, Zinsstag J. Evidence for camels (Camelus bactrianus) as the main intermediate host of Echinococcus granulosus sensu lato G6/G7 in Mongolia. Parasitology Research. 2019 Sep 1;118(9):2583-90.
17. Ohiolei JA, Xia CY, Li L, Liu JZ, Tang WQ, Wu YT, Danqulamu, Zhu GQ, Shi B, Fu BQ, Yin H. Genetic variation of Echinococcus spp. in yaks and sheep in the Tibet Autonomous Region of China based on mitochondrial DNA. Parasites & vectors. 2019 Dec 27;12(1):608.
18. Ohiolei JA, Li L, Yan HB, Fu BQ, Jia WZ. Complete mitochondrial genome analysis confirms the presence of Echinococcus granulosus sensu lato genotype G6 in Nigeria. Infection, Genetics and Evolution. 2020 Oct 1;84:104377.
19. Lazzarini LE, Debiaggi MF, Pianciola LA, Mazzeo ML, Soriano SV, Pierangeli NB. Differentiation of Echinococcus granulosus sensu lato species of human origin from Neuquén, Argentina, by multivariate analysis of rostellar hooks morphometry. Journal of helminthology. 2025 Jul 4;99:e66.
20. Nakao M, McManus DP, Schantz PM, Craig PS, Ito A. A molecular phylogeny of the genus Echinococcus inferred from complete mitochondrial genomes. Parasitology. 2006 May;134(5):713-22.
21. Laurimäe T, Kinkar L, Moks E, Bagrade G, Saarma U. Exploring the genetic diversity of genotypes G8 and G10 of the Echinococcus canadensis cluster in Europe based on complete mitochondrial genomes (13 550–13 552 bp). Parasitology. 2023 Jun;150(7):631-7.
22. Shumuye NA, Li L, Ohiolei JA, Qurishi SA, Li WH, Zhang NZ, Wu YT, Wu YD, Gao SZ, Zhang FH, Tian XQ. Update on the genetic diversity and population structure of Echinococcus granulosus in Gansu Province, Tibet Autonomous Region, and Xinjiang Uygur Autonomous Region, Western China, inferred from mitochondrial cox 1, nad 1, and nad 5 sequences. Parasitology Research. 2023 May;122(5):1107-26.
23. Wassermann M, Addy F, Kokolova L, Okhlopkov I, Leibrock S, Oberle J, Oksanen A, Romig T. High genetic diversity of Echinococcus canadensis G10 in northeastern Asia: is it the region of origin?. Parasitology. 2024 Jan;151(1):93-101.
24. Wu D, Zhang J, Wang J, Zhao Y, Wang L, Mao Q, Lin H, Yao G, Zhang G, Wang X, Xue C. First Case Report of Cystic Echinococcosis Caused by G7 Genotype Echinococcus intermedius Confirmed by Genetic Sequencing—Southern China, December 2023. China CDC Weekly. 2024 Jun 7;6(23):558.
25. Biedermann A, Laurimäe T, Anijalg L, Kamenetzky L, Soriano SV, Pierangeli N, Lazzarini LE, Umhang G, Bold B, Bayasgalan C, Karamon J. Zoonotic Echinococcus granulosus sensu lato genotypes G6 and G7: new insights from the global mitogenome analysis. International Journal for Parasitology. 2025 Apr 16.
26. Laurimäe T, Kinkar L, Romig T, Umhang G, Casulli A, Omer RA, et al. Analysis of nad2 and nad5 enables reliable identification of genotypes G6 and G7 within the species complex Echinococcus granulosus sensu lato. Infection, Genetics and Evolution. 2019 Oct;74:103941. doi:10.1016/j.meegid.2019.103941
