# Supplementary material for: Molecular evidence of Echinococcus canadensis (G6/G7) predominance in Mongolian livestock and its implications for control
Source: PLoS Negl Trop Dis. 2026 Jun 15;20(6):e0014433. doi: 10.1371/journal.pntd.0014433 (PMC13278582; doi:10.1371/journal.pntd.0014433)
Supplement: S5 Table — (DOCX) [file pntd.0014433.s006.docx]

**S5 Table. Haplotype frequency for countries and hosts.**

| **Country** | **Count** |  | **Host Group** | **Count** | **Host** |
| --- | --- | --- | --- | --- | --- |
| Mongolia | 37 |  | Human | 38 | Human: 38 |
| Russia | 27 |  | Sheep | 13 | Sheep: 13 |
| China | 17 |  | Wolf | 12 | Wolf: 12 |
| Argentina | 5 |  | Dog | 12 | Dog: 12 |
| Kazakhstan | 3 |  | Bovine | 6 | Cattle: 3; Buffalo: 2; Yak: 1 |
| Turkey | 3 |  | Cervid | 6 | Moose: 3; Elk: 2; Reindeer: 1 |
| France | 2 |  | Camel | 6 | Camel: 6 |
| Nepal | 1 |  | Goat | 5 | Goat: 5 |
| Armenia | 1 |  | Pig | 4 | Pig: 4 |
| India | 1 |  |  |  |  |
| Algeria | 1 |  |  |  |  |
| Nigeria | 1 |  |  |  |  |
| Pakistan | 1 |  |  |  |  |
| Moldova | 1 |  |  |  |  |
| Iran | 1 |  |  |  |  |
