# Supplementary material for: Molecular evidence of Echinococcus canadensis (G6/G7) predominance in Mongolian livestock and its implications for control
Source: PLoS Negl Trop Dis. 2026 Jun 15;20(6):e0014433. doi: 10.1371/journal.pntd.0014433 (PMC13278582; doi:10.1371/journal.pntd.0014433)
Supplement: Attachment — Submitted filename: Response to Reviewers 2nd.pdf [file pntd.0014433.s010.pdf]

Response to Reviewers

Dear Editors and Reviewers,

Thank you very much for your careful evaluation of our revised manuscript. We are very grateful that the reviewers found the revision improved. The comments and suggestions throughout the review process have greatly helped us to improve the manuscript.

We have addressed the remaining comments from the reviewers, and our point-by-point responses are provided below. We also made a few minor wording changes to improve clarity, which are shown in the tracked-changes version.

Reviewer #1

Comment 1.
The revision was carefully realized, improving the quality of the manuscript, notably clearly identifying some limitations without detracting from the interesting results obtained.

Response:
We sincerely thank the reviewer for this positive assessment.

Comment 2.
Fig. 4 is really good, but the number of genotype should not be written as a superscript but rather normally following the letter G. Please just change this.

Response:
Thank you for pointing this out. We have revised Figure 4, as well as the phylogenetic tree, so that the genotype labels are written normally, for example G6 and G7, rather than as superscript.

Reviewer #2

Comment 1.
All comments have been duly addressed, and the authors have carefully revised and adjusted the manuscript accordingly. I would only like to offer a couple of final minor suggestions.

Response:
We sincerely thank the reviewer for this positive evaluation.

Comment 2.
Please review the use of *sensu lato* and *sensu stricto* and their abbreviations (*s.l.* and *s.s.*): they should be in italics.

Response:
We have reviewed the manuscript throughout and revised the formatting of *sensu lato*, *sensu stricto*, *s.l.*, and *s.s.*, ensuring that they are consistently italicized.

Comment 3.
At lines 514–516, it is appropriate to have added information on control programmes as suggested; however, one or more supporting references should also be cited, e.g. Gemmell MA, Roberts MG, Beard TC, Campano Díaz S, Lawson JR, Nonnemaker JM. Control of *Echinococcus granulosus* in New Zealand, Tasmania and Uruguay. *Veterinary Parasitology*. 2001;96:259–277.

Response:
Thank you for this important suggestion. We have added relevant supporting references to this statement (Lines 512–514, refs. 62 and 63).
