# Supplementary material for: Molecular evidence of Echinococcus canadensis (G6/G7) predominance in Mongolian livestock and its implications for control
Source: PLoS Negl Trop Dis. 2026 Jun 15;20(6):e0014433. doi: 10.1371/journal.pntd.0014433 (PMC13278582; doi:10.1371/journal.pntd.0014433)
Supplement: Attachment — Submitted filename: Response to reviewers.docx [file pntd.0014433.s009.docx]

**Additional Editor Comments:**

**General comment:** Please carefully consider the reviewer feedback and make appropriate revisions

**Response.** We have carefully considered all reviewer comments and revised the manuscript accordingly. A detailed point-by-point response is provided in the Response to Reviewers document. In addition to the revisions made in response to the reviewers’ comments, we made a small number of minor editorial changes throughout the manuscript to improve clarity, consistency, and readability. These changes are visible in the tracked version.

**Journal requirements:**

**Comment 1.**  We ask that a manuscript source file is provided at Revision. Please upload your manuscript file as a .doc, .docx, .rtf or .tex. If you are providing a .tex file, please upload it under the item type u2018LaTeX Source Fileu2019 and leave your .pdf version as the item type u2018Manuscriptu2019.

**Response**. We have uploaded the revised manuscript as a .docx file, as requested.

**Comment 2.** Please upload all main figures as separate Figure files in .tif or .eps format. For more information about how to convert and format your figure files please see our guidelines:

https://journals.plos.org/plosntds/s/figures

**Response.** All main figures have been removed from the manuscript file and uploaded separately. The figure files have been prepared as separate high-resolution image in .tif format files. NAAS tool modified them into required dimensions and resolution.

**Comment 3.** We have noticed that you have uploaded Supporting Information files, but you have not included a list of legends. Please add a full list of legends for your Supporting Information files after the references list.

**Response.** A full list of legends for all Supporting Information files has now been added after the References section in the manuscript. Line 816-826.

**Comment 4.**Some material included in your submission may be copyrighted. According to PLOSu2019s copyright policy, authors who use figures or other material (e.g., graphics, clipart, maps) from another author or copyright holder must demonstrate or obtain permission to publish this material under the Creative Commons Attribution 4.0 International (CC BY 4.0) License used by PLOS journals. Please closely review the details of PLOSu2019s copyright requirements here: PLOS Licenses and Copyright. If you need to request permissions from a copyright holder, you may use PLOS's Copyright Content Permission form. Please respond directly to this email and provide any known details concerning your material's license terms and permissions required for reuse, even if you have not yet obtained copyright permissions or are unsure of your material's copyright compatibility. Once you have responded and addressed all other outstanding technical requirements, you may resubmit your manuscript within Editorial Manager.

Potential Copyright Issues:

- Please confirm (a) that you are the photographer of Figure 2., or (b) provide written permission from the photographer to publish the photo(s) under our CC BY 4.0 license.

- Figures 1 and 3. Please (a) provide a direct link to the base layer of the map (i.e., the country or region border shape) and ensure this is also included in the figure legend; and (b) provide a link to the terms of use / license information for the base layer image or shapefile. We cannot publish proprietary or copyrighted maps (e.g. Google Maps, Mapquest) and the terms of use for your map base layer must be compatible with our CC BY 4.0 license.

**Response.** **For the photographs in Fig2:** we confirm that the photographs in Fig 2 were taken by the first author and co-authors. There was a short communication about this with the PLOS ntd editors briefly before the submission. **For the map in Fig 1 and Fig 3:** We have revised the relevant figure legends to include the source, download link, and licensing information for the base map shapefiles used in Figures 1 and 3. This information has been added to the legend of Fig 1 (Lines 185–188) and to the legend of Fig 3 (Lines 376-380). In addition, a sentence describing the map data source and license has been added to the Methods section (Line 273-275).

**Comment 5.** Please amend your detailed Financial Disclosure statement. This is published with the article. It must therefore be completed in full sentences and contain the exact wording you wish to be published.

- State the initials, alongside each funding source, of each author to receive each grant. For example: "This work was supported by the National Institutes of Health (####### to AM; ###### to CJ) and the National Science Foundation (###### to AM)."

- State what role the funders took in the study. If the funders had no role in your study, please state: "The funders had no role in study design, data collection and analysis, decision to publish, or preparation of the manuscript.".

If you did not receive any funding for this study, please simply state: u201cThe authors received no specific funding for this work.u201d

**Response.** Thank you. We have revised the Financial Disclosure statement to provide full-sentence wording with additional detail, as requested. Current disclosure reads as the following: “*This work was supported by the National Natural Science Foundation of China (Grant ID: 82350410494 to BB). The funders had no role in study design, data collection and analysis, decision to publish, or preparation of the manuscript.”*

**Response to Reviewer’s comments**

**Reviewer #1:**

**Comment 1:** This manuscript describes a survey about presence of *E. granulosus* s.l. species at the slaughterhouse in four provinces of Mongolia. From selected cysts supposed to be due to *E. granulosus* s.l. according to morphological characteristics, molecular identification was tried using two mitochondrial targets. A rather low proportion (10%) of cysts samples has succeed to provide DNA sequences, mainly corresponding to *E. canadensis* G6/7 when *E. granulosus* s.s. is generally the main species infecting sheep. The predominance of *E. canadensis* G6/7 in camels, goats but especially sheep confirms the needs of molecular identification in the aim to fight against *E. granulosus* s.l. especially regarding the use of EG95 vaccine.

The objective of the study to identifiy species of *E. granulosus* s.l. was correctly investigated regarding sampling. The number of animals investigated and cysts observed should ensure robust data. No ethical concern as it concerned obtention of samples from dead animals at the slaughterhouse.

**Response:** Thank you for your time and careful review of our manuscript. We appreciate your valuable comments and suggestions, which helped improve the manuscript significantly. We have carefully reviewed all parts of the manuscript in response to the reviewers’ comments and revised it accordingly.

**Comment 2:** Concerning the choice of mitochondrial markers, it is surprising to not have tried to define with confidence if is genotype G6 or G7 by using nad5 and nad2 genes as previously published (Laurimae et al. 2019). This is of importance especially when the objective is to clarify the genotype profile. The short *cox1* and atp6 sequences obtained may be not enough discriminating to be sure between G6 and G7.

**Response:** We thank the reviewer for this valuable suggestion and for highlighting Laurimäe et al. (2019). We agree that higher-resolution mitochondrial markers such as nad2, nad5, or complete mitogenomes can improve lineage discrimination within *E. canadensis*. However, previous studies have shown that mitochondrial divergence between the G6 and G7 lineages is relatively shallow, with only a limited number of diagnostic substitutions. Following the reviewer’s recommendation, we re-analysed our dataset by examining the informative *cox1* nucleotide positions reported by Laurimäe et al. (2019) that overlap with our sequenced fragment. This analysis supported assignment of haplotypes H1–H3 to the G6 lineage and haplotype H4 to the G7a lineage, including concordance at the key diagnostic positions (9656 bp and 9734 bp). We acknowledge that the partial *cox1* fragment does not provide full resolution of all mitochondrial lineages within *E. canadensis*, and genotype inference should therefore be interpreted with appropriate caution. Importantly, this additional subtyping does not affect the primary conclusion of the study, namely the predominance of *E. canadensis* relative to *E. granulosus* sensu stricto (G1/G3), which remains highly relevant for control strategies such as EG95 vaccination planning. Please see the updated Methods (Lines 249-253), Results (Lines 356-359), and throughout discussion, Genotype columns in Table 3 and 4, S4 Table, as well as in Abstract (line 34-35, 41-42)

**Comment 3**: The analyses were reealize as described in materials and methods but the rate of success to obtain DNA sequences is low with only 10% of samples succeeding to obtain DNA sequences and generally only for only one of the two mitochondrial targets. Cacified cysts seems to be largely present and can explain difficulties for PCR amplifications. Did the authors tried to dilute the DNA as there can be some inhibitors or even decrease the annealing temperature to facilitate amplification? It is not supposed that E. granulsous s.s. will have more difficulties to be amplified than *E. canadensis* and the predominance of the latter species is not questionned here.

**Response** : Thank you for this helpful comment. During assay optimisation, using a subset of representative cyst samples, we tested several approaches to improve amplification, including DNA dilution to reduce potential inhibitors, adjustment of annealing temperatures, and changes in PCR reagents. Because many samples were calcified, amplification success remained limited. Lower annealing temperatures increased non-specific amplification, whereas higher temperatures reduced or eliminated amplification in many samples. DNA dilution occasionally produced visible bands, but results were inconsistent and often did not lead to successful Sanger sequencing. We therefore selected the final PCR conditions and reagents that provided the most consistent specific amplification and sequencing performance; the protocol applied to all samples is provided in Table S1. We have now clarified this in the Methods section (Line 237-239).

**Comment 4:** The halpotype networks would me more cleare to interpret regarding genotype determiantion if the reference sequences included were also used to identify two main groups of samples, one corresponding to G6 and the other one to G7.

**Response:** We agree, and we addressed this together with the previous comment on G6 versus G7 discrimination (please see our response to Comment 2). We revised Fig. 4 so that the reference haplotypes and haplotypes we found (H1-H4) are now presented in separate genotype-defined panels: *E. canadensis* (G6), *E. canadensis* (G7), *E. canadensis* (G8/G10), and *E. granulosus* s.s. (G1/G3). Please see the updated Fig 4 and genotype columns in S4 Table.

**Comment 5**: The presence or absence of protoscolex is not mentionned when it is important to evaluate the epidemiological role in the lifecycle of each species.

**Response:** We agree that cyst fertility/viability (e.g., protoscolex presence) is important for interpreting the epidemiological role of different intermediate hosts. We could not conducted this assessment mainly due to our remote setting of the sampling. We have now stated this explicitly in the Methods (Line 201-203) and expanded it in the Limitations (544-548).

**Comment 6:** l. 277: organ localization of cysts is difficult to interepret as we don't know if it *E. granulosus*, T. hydatigena or may be due to bacterial infection ...

**Response:** We agree that organ localization based on gross cystic lesions should be interpreted with caution, as not all lesions were confirmed as *E. granulosus* s.l. To avoid overinterpretation, we removed the organ category from Table 2. Organ distribution is slightly modified. The footnote for organ from the Table 2 is now added to the text (Line 297-301).

**Comment 7**: All figures and tables are relevant and clear excepting: I) haplotype networks with genotype identification as mentionned upper; 2) in table 2, columns "Cysts n (%)" and 95% CI for cysts is not useful. In table 2, columns "Cysts n (%)" and 95% CI for cysts is not useful

**Response:** We have revised Fig 4, Table 3, Table 4, S4 Table regarding the genotype information (please see the responses to Comment 2 and 4). We removed the “Cysts n (%)” and cyst-level 95% CI columns from Table 2 as suggested.

**Comment 8.** The conclusions are mainly supported by the data. nevertheless, there are some limitations as the exact genotype was not determinded while the term genotype is largely used in the manuscript. The use of the current taxonomy of E. granulsous needs to be used, especially not using strain designation.

**Response:** We adjusted the wording throughout using the current terms and removed term “strain”. We have provided current species-level taxonomy in the introduction (Line 113-117).

**Comment 9.** One main point not discussed in the manuscript is the relative role of the different host species in the circulation of *E.canadensis* G6/7. As there is a very high proportion of cacified cysts and no proof of protscolex presence especially in sheep it seems difficult to be sure about the large importance of sheep in the maintain of *E. canadensis* compared to goats and camels.

**Response.** Thank you for this important point. We would like to clarify that it was not our intention to imply that sheep are a main maintenance host for *E. canadensis* in Mongolia. Our main finding is that, among the cysts that could be successfully genotyped, *E. canadensis* (G6/G7) was detected more frequently than *E. granulosus* s.s. (G1/G3), including in sheep from the surveyed provinces. We also agree that this result alone is not sufficient to interpret the relative role of host species in transmission, as we did not assess the presence of protoscoleces or overall cyst fertility/viability. We observed that many cystic lesions were small and calcified. This raises the possibility that a substantial proportion of *E. canadensis* infections in sheep (since most samples are from sheep) may be non-fertile or degenerating, which could indicate a more limited contribution of sheep to transmission. We added a section to the Discussion (lines 488–490) on the role of sheep and expanded the Limitations section (lines 546–548) to further elaborate the study’s limitations in determining the role of host animals.

**Comment 10 (summary comment)**.This paper would be useful for a better understanding of the epidemiological situation about *E. granulosus* s.l. in Mongolia, nevertheless there are two main points to take into consideration.

- there is no accurate genotype determination between G6 and G7:

- need to evaluate relative contribution of the different host species (goat, sheep, camel):

**Response.** Thank you for this helpful overall assessment of our manuscript and for highlighting these two important points. We fully agree that accurate G6/G7 discrimination and the relative contribution of different host species are important considerations when interpreting our findings. The first point, regarding genotype discrimination between G6 and G7, has been addressed in our responses to Comments 2 and 4. Regarding the second point, we acknowledge that the relative contribution of sheep, goats, and camels to transmission cannot be determined from the present data without direct assessment of cyst fertility/viability, including protoscolex presence. This was beyond the main scope of the current study, which was primarily aimed at characterizing the genetic profile of *E. granulosus* s.l. in livestock to inform control planning. We have therefore clarified this limitation in the Methods, Discussion, and Limitations sections and have recommended that future larger-scale studies include fertility assessment in order to better evaluate the transmission role of different host species (please see our response to Comments 9 and 5).

**Reviewer #2:**

**General Assessment**

**Comment 1.** The manuscript provides valuable new data on the molecular epidemiology of CE lesions in intermediate hosts in Mongolia. The sampling effort is substantial and well supported by the dataset. The findings complement previously available information, often more fragmented, and contribute to discussions on the potential role of vaccination in control programs, as well as establishing a baseline for monitoring temporal trends in Eg s.l. genetic variants. However, several methodological and interpretative issues require clarification before the manuscript can be considered for publication.

**Response.** Thank you very much for your positive overall assessment of our manuscript and for recognizing its contribution to the molecular epidemiology of CE in Mongolia. We sincerely appreciate the valuable time you devoted to the thorough and rigorous review of our manuscript, and we are grateful for your constructive comments, important suggestions, and meticulous attention to minor errors. We have revised all the comments point by point.

**Major Comments**

**Comment 2.** High number of non amplified samples
A large proportion of samples suggestive of CE failed to amplify. The authors attribute this to cyst condition (calcified, lacking fluid), yet the statistical analysis, although limited, does not support this explanation. Fresher samples did not show higher amplification success.
The authors should therefore consider and discuss additional possible causes:
• (a) Not all samples may belong to *E. granulosus* s.l., as evidenced by the identification of T. hydatigena in some specimens.
• (b) Potential limitations in DNA extraction and amplification protocols.

**Response:** Thank you. We agree that the overall genotyping success was low and that cyst condition alone may not fully explain this pattern. We have therefore revised the manuscript to describe the low amplification/sequencing success as likely multifactorial, including: (a) not all gross cystic lesions representing *E. granulosus* s.l., as supported by the identification of *T. hydatigena* in a subset; (b) degenerated or calcified lesions containing limited parasite tissue and possible PCR inhibitors; and (c) potential limitations in DNA extraction and amplification protocols. During assay optimisation, we tested several approaches to improve performance and selected the protocol that provided the most consistent results in S1 Table. Corresponding revisions were made in the Discussion (496-506).

**Comment 3:** Importantly, the authors do not report the use of positive controls in either protocol. This omission prevents verification of reaction performance. The implications of points (a) and (b) must be clearly addressed.

**Response:** Thank you for noticing this point. We clarify in the Methods (Line 239-240) that positive controls and no-template controls were included in every PCR run. This information was omitted by mistake from the submitted version and has now been added to improve transparency and reproducibility of the molecular procedures.

**Comment 4.** Interpretation of “globally dominant” G1–G3 haplotypes
Throughout the manuscript, the authors imply that the G1–G3 haplotype identified in this study is “globally dominant,” contrasted with G6/G7 haplotypes, which are described as having a more local genetic signature.
This wording is misleading. If the authors intend to state that G1–G3 is globally dominant in general, this should be clarified. As currently written, it suggests that the specific G1–G3 haplotype found in this dataset is globally predominant, which is not supported by the haplotype networks. Indeed, G6/G7 haplotypes are not genetically distant from isolates from Argentina or Moldova.
A clearer and more accurate interpretation is needed.

**Response:** We agree that our wording was unclear and could be interpreted as suggesting that the specific G1/G3 haplotype detected in our dataset (H5) is globally predominant. This was not our intention. Rather, we intended to refer to *E. granulosus* s.s. (G1/G3) as a group that is widely reported as a common cause of cystic echinococcosis, rather than to claim global predominance of the single haplotype identified in this study. We have revised the text throughout the manuscript to clarify this distinction, removed wording such as “globally dominant,” and avoided implying that G6/G7 has a more local distribution than G1/G3. Revision in Line 476-478 in Discussion.

**Comment 5.** Haplotype coding in the table (*cox1* vs *atp6 II*)
comment: The table lists haplotypes for both *cox1* and *atp6 II* using progressive codes (H1, H2, H3…). However, in the text the authors refer exclusively to *cox1* haplotypes, which may cause confusion.
To improve clarity, the authors should adopt one of the following solutions:
• Option 1: Always specify “*cox1* H1”, “*cox1* H2”, etc.
• Option 2 (preferred): Use a different coding system for *atp6 II* haplotypes to avoid overlap.

**Response:** We acknowledge that naming both as H might be confusing. To improve clarity and avoid confusion between loci, we have adopted a separate coding system for *ATP6 II* haplotypes (HA1–HA3), while maintaining the H1–H5 nomenclature for *COX1*. Table 3 and the corresponding Results (359-370) text have been revised accordingly.

**Comments for editorial and data presentation**

We really appreciate the reviewer 2 for providing this highly helpful corrections and suggestions. We revised them all accordingly and provided the new line numbers.

**Abstract/Authors summary**

**Comment 6:** Line 64: “Echinococcus types” – it would be more accurate to refer to species/genotypes rather than “types”.

**Response:** Corrected, new line number 65

**Comment 7:** Line 65: *E. canadensis* should be italicized.

**response:** Corrected, new line number 66

**Comment 8**. Line 68: A period appears to be missing at the end of the sentence.

**Response:** This sentence was removed from the Author Summary due to the change in the manuscript.

**Introduction**

**Comment 9**: Line 83: Consider rephrasing as “eggs shed through feces”.

**Response:** Corrected, new line number 85

**Comment 10.** Line 86: The sentence seems incomplete; perhaps a verb such as “infested with” is missing.

**Response:** Revised the text for clarity, new line number 88.

**Comment 11:** Line 86: Larval stages may contain protoscoleces.

**Response:** Revised the text, new line number 88-89.

**Comment 12**. Line 106: Please add “also Chile”.

**Response:** Revised the text with adding Chile, the new line number 108.

**Comment 13**: Line 111: The statement about “ten species/genotypes” is not fully accurate. For example, G2 and G9 are no longer widely recognized. It would be helpful if the authors could briefly clarify the taxonomy and epidemiology of the *E. granulosus* sensu lato complex (species, hosts, distribution). The currently accepted nomenclature includes: *E. granulosus* s.s. (G1 and G3), E. equinus (G4), E. ortleppi (G5), *E. canadensis* (G6/G7 cluster), and *E. canadensis* (G8 and G10).

**Response.** Thank you for this helpful point. We have revised the Introduction and Abstract to align terminology with the currently accepted taxonomy of the *Echinococcus granulosus* sensu lato complex. While mitochondrial genotypes (G1–G10) were historically used to describe diversity within the complex, current nomenclature increasingly refers to species-level taxa, including *E. granulosus* sensu stricto (G1/G3), *E. equinus* (G4), *E. ortleppi* (G5), and *E. canadensis* (G6/G7 cluster and G8/G10). These revisions have been incorporated to improve taxonomic accuracy and clarity (line 113-117).

**Comment 14**: Line 138: Do the authors have an estimate of CE prevalence in sheep or other livestock species?

**Response**: Unfortunately, robust nationwide estimates of cystic echinococcosis prevalence in livestock in Mongolia are currently lacking, and even localized surveys remain rare. The limited available data are derived from a small number of abattoir-based studies, which limits comparability across regions and host species (128-131).

**Methods**

**Comment 15:** Line 218: Could the authors clarify why only the cyst content was used, and not the cyst wall itself? Additionally, how was the material homogenized?

**Response.** Thank you for this question. DNA was extracted from parasite-derived material, including cyst contents (calcified tissues) and germinal layers. The host-derived fibrous capsule was excluded. Samples were first cut into small pieces using sterile scissors and then mechanically homogenized with a handheld motorized tissue homogenizer prior to Proteinase K digestion and DNA extraction. These details have now been added to the Methods section to improve reproducibility. Please see Lines: 225-227.

**Comment 16**: Line 241: “Taenia” should be capitalized as it begins the sentence.

**Response:** Corrected the text, new line number 258

**Results/Discussion**

**Comment 17.** Line 268: It may be helpful to discuss the differences among provinces in a later section.

**Response:** The Results section has been reorganized so that overall prevalence patterns and molecular confirmation are presented first, followed by province-level comparisons. This restructuring improves clarity and facilitates interpretation of geographic variation (Line 278-293).

**Comment 18**: Lines 213–213 (312): The note “Rooted with V. mustelae; non-Echinococcus references included” seems redundant and may not need to be repeated.

**Response:** Revised the text and removed the redundant phrase. The new line number is 337.

**Comment 19.** Figure S1: – For the *E. canadensis* group, please consider adding “G6/G7 cluster” to the label.

**Response:** We have revised it accordingly in the S1 Fig by adding “G6/G7 cluster”

**Comment 20**. Earlier in the manuscript the authors state that *T. hydatigena* data were not considered as they were beyond the study’s scope; however, they appear in the phylogenetic tree. Since they are included in the analysis, this should be briefly acknowledged in the text.

**Response:** Thank you for noting this. Although *T. hydatigena* was not a primary focus of the study, sequences identified from a subset of lesions were included in the phylogenetic analysis to confirm species identity and illustrate lesion heterogeneity. This has now been briefly acknowledged in the revised text, and the corresponding results are presented in S1 Fig. (Lines 341–346).

**Comment 21**. Line 321: Please also report the detection of distinct *atp6 II* haplotypes.

**Response**. We thank the reviewer for highlighting this omission. We have now added a dedicated section in the Results describing the atp6 haplotype analysis, including the number and distribution of distinct haplotypes detected in the dataset (Line 359-370).

**Comment 22.** Lines 327–328: Highlighting genetic distances between haplotypes may be redundant, as *E. granulosus* s.s. and *E. canadensis* are nearly distinct species.

**Response.** We revised a text in the Result (354-356) and retained only within-cluster distances among H1–H4, which are informative for diversity within the *E. canadensis* group.

**Comment 23. (Question):** Was any sample positive for both *atp6 II* and *cox1*?

**Response.** In this dataset, no cyst yielded sequences for both *COX1* and *ATP6 II*; each successfully genotyped cyst produced a sequence for only one locus. This likely reflects low parasite DNA yield and variable lesion quality, particularly in calcified or degenerated samples collected under abattoir conditions. We have now stated this explicitly in the Results (341).

**Comment 24.** Line 334: Why “a” (singular) sheep? Table 3 indicates that H2 was found in four sheep and one goat. Line 337: More precisely: “in one sheep and one goat”.

**Response:** Thank you for catching this. We corrected the Result text to match Table 3: *COX1* haplotype H2 was detected in four sheep and one goat in our study. Line 382

**Comment 25.** Line 342: “Cluster around” may not be the best expression, as “cluster” implies concentration.

**Response.** Revised the text, new line number 391 in Result

**Comment 26:** Line 347: Replace “in sheep sequence” with “in one sheep”.

**Response:** Revised the text, new line number 403-404 in Result.

**Comment 27.** Line 365. Table 4 could be more informative. For example, for haplotype H4 (suggested to reflect transboundary transmission with China), the table lists previous human and dog cases, but their geographic origin is unclear. Were these from Mongolia, China, or elsewhere? It would be helpful to reference the studies from which these data were derived. This information is not directly evident from Tables S4 and S4.

**Response:** Thank you for this helpful comment. To improve clarity, Table 4 has been revised to explicitly indicate that previously reported host records refer to cases documented in Mongolia. We have also added appropriate source references and revised the table title to ensure accurate interpretation. Please see the new Table 4.

**Comment 28.** Line 373: “*cox1* haplotypes”.

**Response.** Revised the text, new line number 423.

**Comment 29.** Lines 373–374: “a cluster where H2 is central”.

**Response.** Revised the text, new line number 424-425.

**Comment 30.** Lines 374–375: The meaning is unclear. Both G1–G3 and G6/G7 clusters are widespread; see major comments.

**Response**. We have revised it. The detailed answer is in the response to Comment 4. Additionally, the mentioned line for this comment has removed (Line 425).

**Comments 31 for the paragraph from line 383-389:**

- Line 383: Please revise the syntax to improve clarity.

- Lines 384–385: This sentence would also benefit from rephrasing for clarity.

- Lines 385–386: Same suggestion: consider revising for clarity.

- Line 389: Consider adding “for spreading CE” or “to sustain the *E. granulosus* cycle”.

- Line 389: Add the verb “is”.

**Response:** These lines between 383 - 389 in the old manuscript are together one paragraph. We understand that the original text may have been unclear or confusing. We therefore revised the entire paragraph, which now appears in Lines 429-443 of the revised manuscript.

**Comment 32.** Line 403: “Multiple genotypes” – please clarify whether this refers to *E. granulosus* s.s. (G1 and G3) and *E. canadensis* (G6/G7).

**Response.** Revised the text, new line number 452.

**Comment 33.** Line 406: Consider rephrasing as either “G6/G7 was still much more frequent than G1–G3” or “G6/G7 infections were still much more frequent than G1–G3 infections”.

**Response.** Revised the text, new line number 457.

**Comment 34.** Line 412: Add “haplotype” after H2.

**Response.** Corrected, new line number 463.

**Comment 35.** Line 415: “circulate in”.

**Response.** Corrected, new line number 466

**Comment 36**. Line 422: Same concern regarding the representation of G1–G3 vs G6/G7 (see major comments and lines 374–375).

**Response.** We agree. Please see the detailed answer is in the Comment 4, as well as 30. The line 473 (previously 422) is also revised.

**Comment 37**. Line 443: It would be more accurate to state that the amplification rate (prior to sequencing) was low.

**Response.** Revised the text, new line number 496.

**Comment 38**. Line 443: However, this interpretation does not fully align with the statistical results; see major comments.

**Response.** We have revised it and have addressed it in the response to Comment 2 (major comment). The corresponding revision is between line 496 and 506.

**Comment 39.** Line 450: “DNA yield”.

**Response.** Revised the text, new line number 506.

**Comment 40.** Line 458: It may be worth mentioning that in some control programs T. hydatigena lesions are used as a proxy for CE infection in livestock.

**Response**. Revised the text, new line number 514-516.

**Comment 41.** Lines 489–490: This refers to routine visual inspection; therefore, it may not be appropriate to frame it as a limitation.

**Response.** Revised the text, new line number 544-546.

**Comment 42.** Line 506: Since Eg95 vaccination is not applicable due to the predominance of *E. canadensis*, the discussion should focus on alternative control strategies.

**Response.** thank you for this suggestion. We made revision in the final paragraph. Line 565-568. Similar revision was also made in the conclusion of the Abstract : Line 48-50, Authors summary 70-72.

**Comment 43.** Final paragraph: The conclusion is somewhat repetitive. It may be more useful to highlight actionable suggestions, such as establishing prevalence baselines for long-term monitoring, or noting that certain host categories (e.g., camels in UG) may be at higher risk of infection.

**Response.** Thank you for this important suggestion. We have revised our final paragraph, changed some repetitiveness and added more practical recommendation such further camel study in southern province, improve surveillance system for baseline data, and cross border survey: respected revisions are in line 562-563; 568-570; 572-573;
