# Supplementary material for: Molecular evidence of Echinococcus canadensis (G6/G7) predominance in Mongolian livestock and its implications for control
Source: PLoS Negl Trop Dis. 2026 Jun 15;20(6):e0014433. doi: 10.1371/journal.pntd.0014433 (PMC13278582; doi:10.1371/journal.pntd.0014433)
Supplement: Peer review history

# Peer review: Decision Letter 0

**DOI:** 10.1371/journal.pntd.0014433.r001

**Authors:** Peter Fischer, Bruce Rosa

26 Jan 2026

PNTD-D-25-02313

Molecular evidence of Echinococcus canadensis (G6/G7) predominance in Mongolian livestock and its implications for control

PLOS Neglected Tropical Diseases

Dear Dr. Bold,

Thank you for submitting your manuscript to PLOS Neglected Tropical Diseases. After careful consideration, we feel that it has merit but does not fully meet PLOS Neglected Tropical Diseases's publication criteria as it currently stands. Therefore, we invite you to submit a revised version of the manuscript that addresses the points raised during the review process.

Please submit your revised manuscript within by Mar 27 2026 11:59PM. If you will need more time than this to complete your revisions, please reply to this message or contact the journal office at plosntds@plos.org. When you're ready to submit your revision, log on to https://www.editorialmanager.com/pntd/ and select the 'Submissions Needing Revision' folder to locate your manuscript file.

Please include the following items when submitting your revised manuscript:

* A letter that responds to each point raised by the editor and reviewer(s). You should upload this letter as a separate file labeled 'Response to Reviewers'. This file does not need to include responses to any formatting updates and technical items listed in the 'Journal Requirements' section below.

* A marked-up copy of your manuscript that highlights changes made to the original version. You should upload this as a separate file labeled 'Revised Manuscript with Track Changes'.

* An unmarked version of your revised paper without tracked changes. You should upload this as a separate file labeled 'Manuscript'.

If you would like to make changes to your financial disclosure, competing interests statement, or data availability statement, please make these updates within the submission form at the time of resubmission. Guidelines for resubmitting your figure files are available below the reviewer comments at the end of this letter.

We look forward to receiving your revised manuscript.

Kind regards,

Bruce A. Rosa

Academic Editor

PLOS Neglected Tropical Diseases

Peter Fischer

Section Editor

PLOS Neglected Tropical Diseases

Shaden Kamhawi

co-Editor-in-Chief

PLOS Neglected Tropical Diseases

orcid.org/0000-0003-4304-636XX

Paul Brindley

co-Editor-in-Chief

PLOS Neglected Tropical Diseases

orcid.org/0000-0003-1765-0002

**Additional Editor Comments:**

Please carefully consider the reviewer feedback and make appropriate revisions.

**Journal Requirements:**

1) We ask that a manuscript source file is provided at Revision. Please upload your manuscript file as a .doc, .docx, .rtf or .tex. If you are providing a .tex file, please upload it under the item type u2018LaTeX Source Fileu2019 and leave your .pdf version as the item type u2018Manuscriptu2019.

2) Please upload all main figures as separate Figure files in .tif or .eps format. For more information about how to convert and format your figure files please see our guidelines:

https://journals.plos.org/plosntds/s/figures

3) We have noticed that you have uploaded Supporting Information files, but you have not included a list of legends. Please add a full list of legends for your Supporting Information files after the references list.

4) Some material included in your submission may be copyrighted. According to PLOSu2019s copyright policy, authors who use figures or other material (e.g., graphics, clipart, maps) from another author or copyright holder must demonstrate or obtain permission to publish this material under the Creative Commons Attribution 4.0 International (CC BY 4.0) License used by PLOS journals. Please closely review the details of PLOSu2019s copyright requirements here: PLOS Licenses and Copyright. If you need to request permissions from a copyright holder, you may use PLOS's Copyright Content Permission form.

Please respond directly to this email and provide any known details concerning your material's license terms and permissions required for reuse, even if you have not yet obtained copyright permissions or are unsure of your material's copyright compatibility. Once you have responded and addressed all other outstanding technical requirements, you may resubmit your manuscript within Editorial Manager.

Potential Copyright Issues:

- Please confirm (a) that you are the photographer of Figure 2., or (b) provide written permission from the photographer to publish the photo(s) under our CC BY 4.0 license.

- Figures 1 and 3. Please (a) provide a direct link to the base layer of the map (i.e., the country or region border shape) and ensure this is also included in the figure legend; and (b) provide a link to the terms of use / license information for the base layer image or shapefile. We cannot publish proprietary or copyrighted maps (e.g. Google Maps, Mapquest) and the terms of use for your map base layer must be compatible with our CC BY 4.0 license.

Note: if you created the map in a software program like R or ArcGIS, please locate and indicate the source of the basemap shapefile onto which data has been plotted.

If your map was obtained from a copyrighted source please amend the figure so that the base map used is from an openly available source. Alternatively, please provide explicit written permission from the copyright holder granting you the right to publish the material under our CC BY 4.0 license.

If you are unsure whether you can use a map or not, please do reach out and we will be able to help you. The following websites are good examples of where you can source open access or public domain maps:

* U.S. Geological Survey (USGS) - All maps are in the public domain. (http://www.usgs.gov)

* PlaniGlobe - All maps are published under a Creative Commons license so please cite u201cPlaniGlobe, http://www.planiglobe.com, CC BY 2.0u201d in the image credit after the caption. (http://www.planiglobe.com/?lang=enl)

* Natural Earth - All maps are public domain. (http://www.naturalearthdata.com/about/terms-of-use/).

5) Please amend your detailed Financial Disclosure statement. This is published with the article. It must therefore be completed in full sentences and contain the exact wording you wish to be published.

- State the initials, alongside each funding source, of each author to receive each grant. For example: "This work was supported by the National Institutes of Health (####### to AM; ###### to CJ) and the National Science Foundation (###### to AM)."

- State what role the funders took in the study. If the funders had no role in your study, please state: "The funders had no role in study design, data collection and analysis, decision to publish, or preparation of the manuscript.".

If you did not receive any funding for this study, please simply state: u201cThe authors received no specific funding for this work.u201d

**Reviewers' Comments:**

Reviewer's Responses to Questions

**Key Review Criteria Required for Acceptance?**

As you describe the new analyses required for acceptance, please consider the following:

**Methods**

-Are the objectives of the study clearly articulated with a clear testable hypothesis stated?

-Is the study design appropriate to address the stated objectives?

-Is the population clearly described and appropriate for the hypothesis being tested?

-Is the sample size sufficient to ensure adequate power to address the hypothesis being tested?

-Were correct statistical analysis used to support conclusions?

-Are there concerns about ethical or regulatory requirements being met?

Reviewer #1: This manuscript describes a survey about presence of E. granulosus s.l. species at the slaughterhouse in four provinces of Mongolia. From selected cysts supposed to be due to E. granulosus s.l. according to morphological characteristics, molecular identification was tried using two mitochondrial targets. A rather low proportion (10%) of cysts samples has succeed to provide DNA sequences, mainly corresponding to E. canadensis G6/7 when E. granulosus s.s. is generally the main species infecting sheep. The predominance of E. canadensis G6/7 in camels, goats but especially sheep confirms the needs of molecular identification in the aim to fight against E. granulosus s.l. especially regarding the use of EG95 vaccine.

The objective of the study to identifiy species of E. granulosus s.l. was correctly investigated regarding sampling. The number of animals investigated and cysts observed should ensure robust data.

No ethical concern as it concerned obtention of samples from dead animals at the slaughterhouse.

Concerning the choice of mitochondrial markers, it is surprising to not have tried to define with confidence if is genotype G6 or G7 by using nad5 and nad2 genes as previously published (Laurimae et al. 2019). This is of importance especially when the objective is to clarify the genotype profile. The short cox1 and atp6 seqeucnes obtained may be not enough discrimianting to be sure between G6 and G7.

Reviewer #2: (No Response)

**********

**Results**

-Does the analysis presented match the analysis plan?

-Are the results clearly and completely presented?

-Are the figures (Tables, Images) of sufficient quality for clarity?

Reviewer #1: The analyses were reealize as described in materials and methods but the rate of success to obtain DNA sequences is low with only 10% of samples succeeding to obtain DNA sequences and generally only for only one of the two mitochondrial targets. Cacified cysts seems to be largely present and can explain difficulties for PCR amplifications. Did the authors tried to dilute the DNA as there can be some inhibitors or even decrease the annealing temperature to facilitate amplification? It is not supposed that E. granulsous s.s. will have more difficulties to be amplified than E. canadensis and the predominance of the latter species is not questionned here.

The halpotype networks would me more cleare to interpret regarding genotype determiantion if the reference sequences included were also used to identify two main groups of samples, one corresponding to G6 and the other one to G7.

The presence or absence of protoscolex is not mentionned when it is important to evaluate the epidemiological role in the lifecycle of each species.

l. 277: organ localization of cysts is difficult to interepret as we don't know if it E. granulosus, T. hydatigena or may be due to bacterial infection ...

All figures and tables are relevant and clear excepting:

- haplotype networks with genotype identification as mentionned upper.

- in table 2, columns "Cysts n (%)" and 95% CI for cysts is not useful

Reviewer #2: (No Response)

**********

**Conclusions**

-Are the conclusions supported by the data presented?

-Are the limitations of analysis clearly described?

-Do the authors discuss how these data can be helpful to advance our understanding of the topic under study?

-Is public health relevance addressed?

Reviewer #1: The conclusions are mainly supported by the data. nevertheless, there are some limitations as the exact genotype was not determinded while the term genotype is largely used in the manuscript. The use of the current taxonomy of E. granulsous needs to be used, especially not using strain designation.

One main point not discussed in the manuscript is the relative role of the different host species in the circulation of E.canadensis G6/7. As there is a very high proportion of cacified cysts and no proof of protscolex presence especially in sheep it seems difficult to be sure about the large importance of sheep in the maintain of E. canadensis compared to goats and camels.

Reviewer #2: (No Response)

**********

**Editorial and Data Presentation Modifications?**

Use this section for editorial suggestions as well as relatively minor modifications of existing data that would enhance clarity. If the only modifications needed are minor and/or editorial, you may wish to recommend “Minor Revision” or “Accept”.

Reviewer #1: /

Reviewer #2: • Line 64: “Echinococcus types” – it would be more accurate to refer to species/genotypes rather than “types”.

• Line 65: E. canadensis should be italicized.

• Line 68: A period appears to be missing at the end of the sentence.

• Line 83: Consider rephrasing as “eggs shed through feces”.

• Line 86: The sentence seems incomplete; perhaps a verb such as “infested with” is missing.

• Line 86: Larval stages may contain protoscoleces.

• Line 106: Please add “also Chile”.

• Line 111: The statement about “ten species/genotypes” is not fully accurate. For example, G2 and G9 are no longer widely recognized. It would be helpful if the authors could briefly clarify the taxonomy and epidemiology of the E. granulosus sensu lato complex (species, hosts, distribution). The currently accepted nomenclature includes: E. granulosus s.s. (G1 and G3), E. equinus (G4), E. ortleppi (G5), E. canadensis (G6/G7 cluster), and E. canadensis (G8 and G10).

• Line 138: Do the authors have an estimate of CE prevalence in sheep or other livestock species?

• Methods

• Line 218: Could the authors clarify why only the cyst content was used, and not the cyst wall itself? Additionally, how was the material homogenized?

• Line 241: “Taenia” should be capitalized as it begins the sentence.

• Line 268: It may be helpful to discuss the differences among provinces in a later section.

• Lines 213–213: The note “Rooted with V. mustelae; non-Echinococcus references included” seems redundant and may not need to be repeated.

• Figure S1: – For the E. canadensis group, please consider adding “G6/G7 cluster” to the label. – Earlier in the manuscript the authors state that T. hydatigena data were not considered as they were beyond the study’s scope; however, they appear in the phylogenetic tree. Since they are included in the analysis, this should be briefly acknowledged in the text.

• Results / Discussion

• Line 321: Please also report the detection of distinct atp6 II haplotypes.

• Lines 327–328: Highlighting genetic distances between haplotypes may be redundant, as E. granulosus s.s. and E. canadensis are nearly distinct species.

• Question: Was any sample positive for both atp6 II and cox1?

• Line 334: Why “a” (singular) sheep? Table 3 indicates that H2 was found in four sheep and one goat.

• Line 337: More precisely: “in one sheep and one goat”.

• Line 342: “Cluster around” may not be the best expression, as “cluster” implies concentration.

• Line 347: Replace “in sheep sequence” with “in one sheep”.

• Line 365: Table 4 could be more informative. For example, for haplotype H4 (suggested to reflect transboundary transmission with China), the table lists previous human and dog cases, but their geographic origin is unclear. Were these from Mongolia, China, or elsewhere? It would be helpful to reference the studies from which these data were derived. This information is not directly evident from Tables S3 and S4.

• Line 373: “cox1 haplotypes”.

• Lines 373–374: “a cluster where H2 is central”.

• Lines 374–375: The meaning is unclear. Both G1–G3 and G6/G7 clusters are widespread; see major comments.

• Line 383: Please revise the syntax to improve clarity.

• Lines 384–385: This sentence would also benefit from rephrasing for clarity.

• Lines 385–386: Same suggestion: consider revising for clarity.

• Line 389: Consider adding “for spreading CE” or “to sustain the E. granulosus cycle”.

• Line 389: Add the verb “is”.

• Line 403: “Multiple genotypes” – please clarify whether this refers to E. granulosus s.s. (G1 and G3) and E. canadensis (G6/G7).

• Line 406: Consider rephrasing as either “G6/G7 was still much more frequent than G1–G3” or “G6/G7 infections were still much more frequent than G1–G3 infections”.

• Line 412: Add “haplotype” after H2.

• Line 415: “circulate in”.

• Line 422: Same concern regarding the representation of G1–G3 vs G6/G7 (see major comments and lines 374–375).

• Line 443: It would be more accurate to state that the amplification rate (prior to sequencing) was low.

• Line 443: However, this interpretation does not fully align with the statistical results; see major comments.

• Line 450: “DNA yield”.

• Line 458: It may be worth mentioning that in some control programs T. hydatigena lesions are used as a proxy for CE infection in livestock.

• Lines 489–490: This refers to routine visual inspection; therefore, it may not be appropriate to frame it as a limitation.

• Line 506: Since Eg95 vaccination is not applicable due to the predominance of E. canadensis, the discussion should focus on alternative control strategies.

• Final paragraph: The conclusion is somewhat repetitive. It may be more useful to highlight actionable suggestions, such as establishing prevalence baselines for long-term monitoring, or noting that certain host categories (e.g., camels in UG) may be at higher risk of infection.

**********

**Summary and General Comments**

Use this section to provide overall comments, discuss strengths/weaknesses of the study, novelty, significance, general execution and scholarship. You may also include additional comments for the author, including concerns about dual publication, research ethics, or publication ethics. If requesting major revision, please articulate the new experiments that are needed.

Reviewer #1: This paper would be useful for a better understanding of the epidemiological situation about E. granulosus s.l. in Mongolia, nevertheless there are two main points to take into consideration.

- there is no accurate genotype determination between G6 and G7

- need to evaluate relative contribution of the different host species (goat, sheep, camel)

Reviewer #2: General Assessment

The manuscript provides valuable new data on the molecular epidemiology of CE lesions in intermediate hosts in Mongolia. The sampling effort is substantial and well supported by the dataset. The findings complement previously available information, often more fragmented, and contribute to discussions on the potential role of vaccination in control programs, as well as establishing a baseline for monitoring temporal trends in Eg s.l. genetic variants. However, several methodological and interpretative issues require clarification before the manuscript can be considered for publication.

Major Comments

1. High number of non amplified samples

A large proportion of samples suggestive of CE failed to amplify. The authors attribute this to cyst condition (calcified, lacking fluid), yet the statistical analysis, although limited, does not support this explanation. Fresher samples did not show higher amplification success.

The authors should therefore consider and discuss additional possible causes:

• (a) Not all samples may belong to E. granulosus s.l., as evidenced by the identification of T. hydatigena in some specimens.

• (b) Potential limitations in DNA extraction and amplification protocols.

Importantly, the authors do not report the use of positive controls in either protocol. This omission prevents verification of reaction performance. The implications of points (a) and (b) must be clearly addressed.

2. Interpretation of “globally dominant” G1–G3 haplotypes

Throughout the manuscript, the authors imply that the G1–G3 haplotype identified in this study is “globally dominant,” contrasted with G6/G7 haplotypes, which are described as having a more local genetic signature.

This wording is misleading. If the authors intend to state that G1–G3 is globally dominant in general, this should be clarified. As currently written, it suggests that the specific G1–G3 haplotype found in this dataset is globally predominant, which is not supported by the haplotype networks. Indeed, G6/G7 haplotypes are not genetically distant from isolates from Argentina or Moldova.

A clearer and more accurate interpretation is needed.

3. Haplotype coding in the table (cox1 vs atp6 II)

The table lists haplotypes for both cox1 and atp6 II using progressive codes (H1, H2, H3…). However, in the text the authors refer exclusively to cox1 haplotypes, which may cause confusion.

To improve clarity, the authors should adopt one of the following solutions:

• Option 1: Always specify “cox1 H1”, “cox1 H2”, etc.

• Option 2 (preferred): Use a different coding system for atp6 II haplotypes to avoid overlap.

**********

PLOS authors have the option to publish the peer review history of their article (what does this mean?). If published, this will include your full peer review and any attached files.

If you choose “no”, your identity will remain anonymous but your review may still be made public.

**Do you want your identity to be public for this peer review?** For information about this choice, including consent withdrawal, please see our Privacy Policy.

Reviewer #1: No

Reviewer #2: No

[NOTE: If reviewer comments were submitted as an attachment file, they will be attached to this email and accessible via the submission site. Please log into your account, locate the manuscript record, and check for the action link "View Attachments". If this link does not appear, there are no attachment files.]

**Figure resubmission:** While revising your submission, we strongly recommend that you use PLOS’s NAAS tool (https://ngplosjournals.pagemajik.ai/artanalysis) to test your figure files. NAAS can convert your figure files to the TIFF file type and meet basic requirements (such as print size, resolution), or provide you with a report on issues that do not meet our requirements and that NAAS cannot fix.

After uploading your figures to PLOS’s NAAS tool - https://ngplosjournals.pagemajik.ai/artanalysis, NAAS will process the files provided and display the results in the "Uploaded Files" section of the page as the processing is complete. If the uploaded figures meet our requirements (or NAAS is able to fix the files to meet our requirements), the figure will be marked as "fixed" above. If NAAS is unable to fix the files, a red "failed" label will appear above. When NAAS has confirmed that the figure files meet our requirements, please download the file via the download option, and include these NAAS processed figure files when submitting your revised manuscript.

**Reproducibility:**

To enhance the reproducibility of your results, we recommend that authors of applicable studies deposit laboratory protocols in protocols.io, where a protocol can be assigned its own identifier (DOI) such that it can be cited independently in the future. Additionally, PLOS ONE offers an option to publish peer-reviewed clinical study protocols. Read more information on sharing protocols at https://plos.org/protocols?utm_medium=editorial-email&utm_source=authorletters&utm_campaign=protocols

# Author comment: Author response to Decision Letter 1

**DOI:** 10.1371/journal.pntd.0014433.r002

1 Apr 2026

Attachment Submitted filename: Response to reviewers.docx

# Peer review: Decision Letter 1

**DOI:** 10.1371/journal.pntd.0014433.r003

**Authors:** Peter Fischer, Bruce Rosa, Peter Fischer, Bruce Rosa

27 Apr 2026

PNTD-D-25-02313R1Molecular evidence of Echinococcus canadensis (G6/G7) predominance in Mongolian livestock and its implications for controlPLOS Neglected Tropical DiseasesDear Dr. Bold,Thank you for submitting your manuscript to PLOS Neglected Tropical Diseases. After careful consideration, we feel that it has merit but does not fully meet PLOS Neglected Tropical Diseases's publication criteria as it currently stands. Therefore, we invite you to submit a revised version of the manuscript that addresses the points raised during the review process.Please submit your revised manuscript by May 27 2026 11:59PM. If you will need more time than this to complete your revisions, please reply to this message or contact the journal office at plosntds@plos.org. When you're ready to submit your revision, log on to https://www.editorialmanager.com/pntd/ and select the 'Submissions Needing Revision' folder to locate your manuscript file.Please include the following items when submitting your revised manuscript:* A letter that responds to each point raised by the editor and reviewer(s). You should upload this letter as a separate file labeled 'Response to Reviewers'. This file does not need to include responses to any formatting updates and technical items listed in the 'Journal Requirements' section below.* A marked-up copy of your manuscript that highlights changes made to the original version. You should upload this as a separate file labeled 'Revised Manuscript with Track Changes'.* An unmarked version of your revised paper without tracked changes. You should upload this as a separate file labeled 'Manuscript'.If you would like to make changes to your financial disclosure, competing interests statement, or data availability statement, please make these updates within the submission form at the time of resubmission. Guidelines for resubmitting your figure files are available below the reviewer comments at the end of this letter.We look forward to receiving your revised manuscript.Kind regards,Bruce A. RosaAcademic EditorPLOS Neglected Tropical DiseasesPeter FischerSection EditorPLOS Neglected Tropical Diseases

Shaden Kamhawi

co-Editor-in-Chief

PLOS Neglected Tropical Diseases

orcid.org/0000-0003-4304-636XX

Paul Brindley

co-Editor-in-Chief

PLOS Neglected Tropical Diseases

orcid.org/0000-0003-1765-0002

**Additional Editor Comments:**The reviewers were overall very satisfied with the revisions made in response to their comments, and there only remain a few small issues for revision.**Reviewers' comments:**Reviewer's Responses to Questions

**Key Review Criteria Required for Acceptance?**

As you describe the new analyses required for acceptance, please consider the following:

**Methods:**

-Are the objectives of the study clearly articulated with a clear testable hypothesis stated?

-Is the study design appropriate to address the stated objectives?

-Is the population clearly described and appropriate for the hypothesis being tested?

-Is the sample size sufficient to ensure adequate power to address the hypothesis being tested?

-Were correct statistical analysis used to support conclusions?

-Are there concerns about ethical or regulatory requirements being met?

Reviewer #1: this section was modified in order to integrate several points requested by the reviewers notably regarding protoscolex observation and processing o cyst for DNA extraction.

Reviewer #2: (No Response)

**********

**Results:**

-Does the analysis presented match the analysis plan?

-Are the results clearly and completely presented?

-Are the figures (Tables, Images) of sufficient quality for clarity?

Reviewer #1: the results are now more detailed and clear

Reviewer #2: (No Response)

**********

**Conclusions:**

-Are the conclusions supported by the data presented?

-Are the limitations of analysis clearly described?

-Do the authors discuss how these data can be helpful to advance our understanding of the topic under study?

-Is public health relevance addressed?

Reviewer #1: conclusions are sup^ported by the data notably highlighting that withou evalaution of cysts fertility the role of E. canadensis in sheep cannot be evaluated and required further studies

Reviewer #2: (No Response)

**********

**Editorial and Data Presentation Modifications?**

Use this section for editorial suggestions as well as relatively minor modifications of existing data that would enhance clarity. If the only modifications needed are minor and/or editorial, you may wish to recommend “Minor Revision” or “Accept”.

Reviewer #1: Fig4 is really good but the number of genotype should not be written as a superscript but rather normally following the letter G. please just change this

Reviewer #2: (No Response)

**********

**Summary and General Comments:**

Use this section to provide overall comments, discuss strengths/weaknesses of the study, novelty, significance, general execution and scholarship. You may also include additional comments for the author, including concerns about dual publication, research ethics, or publication ethics. If requesting major revision, please articulate the new experiments that are needed.

Reviewer #1: The revision was carrefyly realized improving the quality of the manuscript notably clearly identifying some limitations without detracting from the interesting results obtained

Reviewer #2: All comments have been duly addressed, and the authors have carefully revised and adjusted the manuscript accordingly. I would only like to offer a couple of final minor suggestions:

Please review the use of sensu lato and sensu stricto and their abbreviations (s.l. and s.s.): they should be in italics.

At lines 514-516, it is appropriate to have added information on control programmes as suggested; however, one or more supporting references should also be cited (e.g. Gemmell MA, Roberts MG, Beard TC, Campano Díaz S, Lawson JR, Nonnemaker JM. Control of Echinococcus granulosus in New Zealand, Tasmania and Uruguay. Veterinary Parasitology. 2001;96:259–277).

**********

PLOS authors have the option to publish the peer review history of their article (what does this mean?). If published, this will include your full peer review and any attached files.

If you choose “no”, your identity will remain anonymous but your review may still be made public.

**Do you want your identity to be public for this peer review?** For information about this choice, including consent withdrawal, please see our Privacy Policy.

Reviewer #1: No

Reviewer #2: No

[NOTE: If reviewer comments were submitted as an attachment file, they will be attached to this email and accessible via the submission site. Please log into your account, locate the manuscript record, and check for the action link "View Attachments". If this link does not appear, there are no attachment files.] **Figure resubmission:** While revising your submission, we strongly recommend that you use PLOS’s NAAS tool (https://ngplosjournals.pagemajik.ai/artanalysis) to test your figure files. NAAS can convert your figure files to the TIFF file type and meet basic requirements (such as print size, resolution), or provide you with a report on issues that do not meet our requirements and that NAAS cannot fix.

After uploading your figures to PLOS’s NAAS tool - https://ngplosjournals.pagemajik.ai/artanalysis, NAAS will process the files provided and display the results in the "Uploaded Files" section of the page as the processing is complete. If the uploaded figures meet our requirements (or NAAS is able to fix the files to meet our requirements), the figure will be marked as "fixed" above. If NAAS is unable to fix the files, a red "failed" label will appear above. When NAAS has confirmed that the figure files meet our requirements, please download the file via the download option, and include these NAAS processed figure files when submitting your revised manuscript. **Reproducibility:**To enhance the reproducibility of your results, we recommend that authors of applicable studies deposit laboratory protocols in protocols.io, where a protocol can be assigned its own identifier (DOI) such that it can be cited independently in the future. Additionally, PLOS ONE offers an option to publish peer-reviewed clinical study protocols. Read more information on sharing protocols at https://plos.org/protocols?utm_medium=editorial-email&utm_source=authorletters&utm_campaign=protocols

# Author comment: Author response to Decision Letter 2

**DOI:** 10.1371/journal.pntd.0014433.r004

7 May 2026

Attachment Submitted filename: Response to Reviewers 2nd.pdf

# Editor report: Decision Letter 2

**DOI:** 10.1371/journal.pntd.0014433.r005

**Authors:** Peter Fischer, Bruce Rosa, Peter Fischer, Bruce Rosa, Peter Fischer, Bruce Rosa

2 Jun 2026

Dear Dr. Bold,

We are pleased to inform you that your manuscript 'Molecular evidence of Echinococcus canadensis (G6/G7) predominance in Mongolian livestock and its implications for control' has been provisionally accepted for publication in PLOS Neglected Tropical Diseases.

Before your manuscript can be formally accepted you will need to complete some formatting changes, which you will receive in a follow up email. A member of our team will be in touch with a set of requests.

Please note that your manuscript will not be scheduled for publication until you have made the required changes, so a swift response is appreciated.

IMPORTANT: The editorial review process is now complete. PLOS will only permit corrections to spelling, formatting or significant scientific errors from this point onwards. Requests for major changes, or any which affect the scientific understanding of your work, will cause delays to the publication date of your manuscript.

Should you, your institution's press office or the journal office choose to press release your paper, you will automatically be opted out of early publication. We ask that you notify us now if you or your institution is planning to press release the article. All press must be co-ordinated with PLOS.

Thank you again for supporting Open Access publishing; we are looking forward to publishing your work in PLOS Neglected Tropical Diseases.

Best regards,

Peter U Fischer

Section Editor

PLOS Neglected Tropical Diseases

Peter Fischer

Section Editor

PLOS Neglected Tropical Diseases

Shaden Kamhawi

co-Editor-in-Chief

PLOS Neglected Tropical Diseases

orcid.org/0000-0003-4304-636XX

Paul Brindley

co-Editor-in-Chief

PLOS Neglected Tropical Diseases

orcid.org/0000-0003-1765-0002

***********************************************************

Please address the remaining very minor concerns about some text formatting, and adding one reference.

# Editor report: Acceptance letter

**DOI:** 10.1371/journal.pntd.0014433.r006

**Authors:** Peter Fischer, Bruce Rosa, Peter Fischer, Bruce Rosa, Peter Fischer, Bruce Rosa

Dear Dr. Bold,

We are delighted to inform you that your manuscript, "Molecular evidence of Echinococcus canadensis (G6/G7) predominance in Mongolian livestock and its implications for control," has been formally accepted for publication in PLOS Neglected Tropical Diseases.

We have now passed your article onto the PLOS Production Department who will complete the rest of the publication process. All authors will receive a confirmation email upon publication.

The corresponding author will soon be receiving a typeset proof for review, to ensure errors have not been introduced during production. Please review the PDF proof of your manuscript carefully, as this is the last chance to correct any scientific or type-setting errors. Please note that major changes, or those which affect the scientific understanding of the work, will likely cause delays to the publication date of your manuscript. Note: Proofs for Front Matter articles (Editorial, Viewpoint, Symposium, Review, etc...) are generated on a different schedule and may not be made available as quickly.

Soon after your final files are uploaded, the early version of your manuscript will be published online unless you opted out of this process. The date of the early version will be your article's publication date. The final article will be published to the same URL, and all versions of the paper will be accessible to readers.

For Research Articles, you will receive an invoice from PLOS for your publication fee after your manuscript has reached the completed accept phase. If you receive an email requesting payment before acceptance or for any other service, this may be a phishing scheme. Learn how to identify phishing emails and protect your accounts at https://explore.plos.org/phishing.

Thank you again for supporting open-access publishing; we are looking forward to publishing your work in PLOS Neglected Tropical Diseases.

Best regards,

Shaden Kamhawi

co-Editor-in-Chief

PLOS Neglected Tropical Diseases

Paul Brindley

co-Editor-in-Chief

PLOS Neglected Tropical Diseases
